# Knockout of the *HvCKX1* or *HvCKX3* Gene in Barley (*Hordeum vulgare* L.) by RNA-Guided Cas9 Nuclease Affects the Regulation of Cytokinin Metabolism and Root Morphology

**DOI:** 10.3390/cells8080782

**Published:** 2019-07-26

**Authors:** Sebastian Gasparis, Mateusz Przyborowski, Maciej Kała, Anna Nadolska-Orczyk

**Affiliations:** Department of Functional Genomics, Plant Breeding and Acclimatization Institute–National Research Institute, Radzików, 05-870 Błonie, Poland

**Keywords:** cytokinin, CKX, IPT, barley, CRISPR/Cas9, DEG

## Abstract

Barley is among four of the most important cereal crops with respect to global production. Increasing barley yields to desired levels can be achieved by the genetic manipulation of cytokinin content. Cytokinins are plant hormones that regulate many developmental processes and have a strong influence on grain yield. Cytokinin homeostasis is regulated by members of several multigene families. *CKX* genes encode the cytokinin oxidase/dehydrogenase enzyme, which catalyzes the irreversible degradation of cytokinin. Several recent studies have demonstrated that the RNAi-based silencing of *CKX* genes leads to increased grain yields in some crop species. To assess the possibility of increasing the grain yield of barley by knocking out *CKX* genes, we used an RNA-guided Cas9 system to generate ckx1 and ckx3 mutant lines with knockout mutations in the *HvCKX1* and *HvCKX3* genes, respectively. Homozygous, transgene-free mutant lines were subsequently selected and analyzed. A significant decrease in CKX enzyme activity was observed in the spikes of the ckx1 lines, while in the ckx3 lines, the activity remained at a similar level to that in the control plants. Despite these differences, no changes in grain yield were observed in either mutant line. In turn, differences in CKX activity in the roots between the ckx1 and ckx3 mutants were reflected via root morphology. The decreased CKX activity in the ckx1 lines corresponded to greater root length, increased surface area, and greater numbers of root hairs, while the increased CKX activity in the ckx3 mutants gave the opposite results. RNA-seq analysis of the spike and root transcriptomes revealed an altered regulation of genes controlling cytokinin metabolism and signaling, as well as other genes that are important during seed development, such as those that encode nutrient transporters. The observed changes suggest that the knockout of a single *CKX* gene in barley may be not sufficient for disrupting cytokinin homeostasis or increasing grain yields.

## 1. Introduction

In recent years, efforts taken in cereal breeding programs have focused mainly on increasing resistance to disease and abiotic stress, which is motivated by climate change and decreasing biodiversity. Moreover, little progress has been made toward increasing yield potential. One of the reasons for this limitation is the genetic nature of yield-related traits, which are usually determined by hundreds of quantitative trait loci, and conventional breeding methods are not sufficient for increasing yields to expected levels. Modern biotechnology tools such as genetic transformation, marker-assisted selection, genome editing, and next-generation sequencing can be used as supporting methods in the conventional breeding of crop plants.

Barley (*Hordeum vulgare* L.) is a widely cultivated cereal crop species whose global production is ranked fourth after maize, wheat, and rice (Faostat 2017) [1]. Barley is recognized for its high nutritional value and malting quality, and is used mainly for brewing, distilling, and producing food and feed. Similar to that of the other most important cereal crop species, the progress of increasing the yield potential of barley is unsatisfactory, given the still increasing demand for food production. This problem may be addressed by the identification and utilization of new genes controlling grain size and grain number—the two main components of yield in cereals. Novel genome-editing tools can be particularly useful in these efforts. CRISPR/Cas (clustered regularly interspaced short palindromic repeat/CRISPR associated Cas) is considered a breakthrough technology of genome editing, mainly because of its high effectiveness and technical simplicity. In its basic form, CRISPR/Cas technology can be used for the introduction of targeted knockout mutations at precisely selected genomic locations. In more advanced approaches, this system can also be utilized for the introduction of defined mutations (gene repair), for gene stacking, or for the upregulation or downregulation of gene expression [2]. CRISPR/Cas-based systems have been successfully used for genome editing in barley [3,4,5,6] and other important cereal species [7].

There are several known yield-related genes in barley that could be exploited by genetic engineering to improve plant productivity. These genes are involved in many processes at different developmental stages, including transcriptional regulation, hormone metabolism and signaling, cell division and proliferation, flower formation, and carbohydrate metabolism (reviewed in [8]). Among these genes, the regulators of cytokinin metabolism and signaling are particularly interesting because the implications of cytokinin activity on seed yield have been well established [9]. Cytokinins are plant hormones that are involved in the regulation of a broad spectrum of developmental processes: they stimulate cell division; regulate shoot and root formation; are involved in the growth and development of leaves, flowers, and fruit; and influence seed setting and development [10]. More recent discoveries have revealed the function of cytokinins in biotic and abiotic stress responses [11,12,13]. Cytokinins indicate high physiological activity, and consequently, plants have developed various mechanisms to control the levels of active cytokinin at certain developmental stages. These mechanisms are regulated by the members of several multigene families that are responsible for cytokinin biosynthesis (isopentenyl transferase, *IPT*), activation (LONELY GUY, *LOG*), irreversible degradation (cytokinin oxidase/dehydrogenase, *CKX*), reversible inactivation (zeatin *O*-glucosyltransferase, *ZOG*) and reactivation (β-glucosidase, *GLU*). Cytokinin oxidase/dehydrogenase enzymes (CKX) catalyze cytokinin degradation by cleaving unsaturated isoprenoid side chains from the active cytokinin form. CKX enzymes are encoded by a family of *CKX* genes that show tissue specificity and developmentally dependent patterns of expression [14]. As the key regulators of cytokinin levels, CKX genes have drawn the attention of researchers who have cited their potential for crop improvement because of their manipulation of cytokinin homeostasis. Indeed, several reports have demonstrated that some yield attributes can be improved by the suppression of CKX expression. For instance, the downregulation of *CKX* genes by mutation, RNAi-based silencing, or genome editing led to higher seed numbers and/or seed weight in rice, barley, cotton, and Arabidopsis [15,16,17,18,19,20,21]. 

It has also been demonstrated that *CKX* genes influence root growth and morphology. The overexpression of *CKX* genes and the resulting decrease in cytokinin content led to the increased growth of the root system [22,23]. However, in the case of barley, different phenotypic effects on roots are observed if RNAi or gene knockout techniques are used to suppress the expression of *HvCKX1* [5,16] and *HvCKX3* (this study).

As an alternative approach, cytokinin homeostasis can be manipulated by the overexpression of *IPT* genes. *IPT* belongs to a small family of genes that encode isopentenyl transferases that are directly involved in the de novo synthesis of cytokinin hormones. Similar to *CKX*s, *IPT* genes in barley indicate tissue specificity and development-dependent patterns of expression [23]. The positive effects of ectopic expression of IPT genes on seed yield have been demonstrated in different plant species [9].

Eleven *HvCKX* gene family members have been identified in barley. These genes show various patterns of tissue-specific and temporal expression regulation, which indicate their specialized functions in different plant organs [18,23,24]. In the present study, we used an RNA-guided Cas9 nuclease to generate knockout mutations in the *HvCKX1* and *HvCKX3* genes. According to our knowledge, the biological function of *HvCKX3* has not been studied before in barley using reverse genetics approaches. In wild-type plants, both genes were expressed at the highest level in young spikes between 0–14 DAP (days after pollination) and in seedling roots [18,23,24], i.e., the organs that have a direct or indirect influence on plant yield. The effects of the knockout of *HvCKX1* and *HvCKX3* on the regulation of other genes related to cytokinin metabolism were analyzed at the transcriptomic level. The influence of the knockout mutations on yield attributes as well as root morphology was also evaluated in generated homozygous mutant lines.

## 2. Materials and Methods

### 2.1. Selection of Target Sequences and sgRNA Design

The sequences of the *HvCKX1* (HORVU3Hr1G019850) and *HvCKX3* (HORVU1Hr1G042360) genes were obtained from the Ensembl Plants database. Target sequences that conformed to the G(N)_20_GG or (N)_20_GG template were identified on the sense strand of the coding sequences of both genes (Figure S1 and S2). The potential off-target sequences were searched within the entire genome assembly of cultivar Morex (IBSC v2) using the CasOT program [25] with the following search parameters: -m = single -t =‘ access path to sgRNA sequence in fasta format’ -g = ‘access path to reference genome sequence in fasta format’’ -e = ‘‘access path to the exon annotation of a specific genome in GFT format’ -o = csv, -s = 3, and -n = 5. After validation, the sequence GATCACCGCGGCGTCTCCTACGG, which contains a restriction site for the *Bsm*AI enzyme, was selected for the *HvCKX1* gene, and for the *HvCKX3* gene, the sequence CAAGTTCATCCAGAGCCCCATGG, which contains a restriction site for *Ban*II, was selected. The selected target sequences were verified by PCR amplification and Sanger sequencing in the barley cultivar Golden Promise to ensure that no polymorphism existed between these sequences and the sgRNA. Fragments with consensus sequences to the 20-bp sequence upstream of the PAM (palindromic adjacent motif) motif in the target sequences, were synthesized in the form of complementary oligonucleotides and cloned between the U6 promoter from wheat and the gRNA scaffold in a pCR8/GW/TOPO vector, as described by Gasparis et al. [5]. Two constructs were prepared: one containing sgRNA for *HvCKX1* (ckx1-sgRNA) and another containing sgRNA for *HvCKX3* (ckx3-sgRNA).

### 2.2. Plant Transformation and Selection of Mutant Lines

A binary vector pBract211 [26] containing a synthetic Cas9 gene and Gateway cloning cassette (pBract211-Cas9-GW) was assembled as described previously [5]. The sgRNA sequences assembled in the pCR8/GW/TOPO vector were cloned into the pBract211-Cas9-GW vector via a Gateway reaction using LR Clonase (Invitrogen, Carlsbad, CA, USA) according to the manufacturer’s protocol. Two binary vectors, pBract211-Cas9-ckx1sgRNA and pBract211-Cas9-ckx3sgRNA, were prepared and used independently for the *Agrobacterium*-mediated transformation of immature embryos of the barley cultivar Golden Promise according to the protocol of Harwood et al. [27], with modifications as described by Gasparis et al. [5]. The putative transgenic events were PCR screened for the presence of a T-DNA fragment using hyg405 primers specific to the *hpt* gene (see Table S1 for the primer sequences). For each PCR-positive transgenic plant, leaf tissue samples were collected for the extraction of genomic DNA. The genomic DNA was extracted using the standard CTAB (cetyltrimethylammonium bromide) method [28]. Fragments of the target genes were amplified with specific primers (ckx1-ps and ckx3-ps; see Table S1 for primer sequences) flanking the selected target sequence in *HvCKX1* and *HvCKX3*, respectively, using Q5 Hot-Start Polymerase (New England Biolabs, Frankfurt, Germany). After amplification, 10 μL of the PCR mixture was taken for restriction enzyme digestion with the *Bsm*AI (*HvCKX1*) or *Ban*II (*HvCKX3*) enzyme. The digested amplicons were separated on 1.5% agarose gels and imaged on a Kodak Gel Logic 200 Imaging System. T_0_ plants with the detected mutation were grown until dry mature seeds were collected. Twelve seeds from each T_0_ plant were germinated to select homozygous mutants and null segregants. The germinated seedlings were subsequently screened for the presence of the T-DNA and mutation as described above. PCR products from samples with mutations detected on the gel were cloned into a pGEM-T Easy vector (Promega, Mannheim, Germany) for Sanger sequencing. Then, 10 to 20 clones from each sample were sequenced. Transgene-free, homozygous mutant plants and wild-type null segregants were chosen for further analyses.

### 2.3. CKX Activity Assays

Cytokinin oxidase/dehydrogenase activity was measured in seven DAP spikes of T_1_ plants and in the 10-day-old roots of T_2_ seedlings. The root samples were pooled from six individual seedlings of each T_2_ line. The assay was performed according to the methods of Frebort et al. [29]. Tissue samples were ground to a fine powder in liquid nitrogen and then suspended in a double-excess extraction buffer containing 0.2 M of Tris-HCl (ROTH, Krlsruhe, Germany), 1 mM of phenylmethylsulfonyl fluoride (PMSF) (Sigma-Aldrich, Hamburg, Germany), and 0.3% Triton X-100 (ROTH, Krlsruhe, Germany ) (pH 8.0). Tissue debris was removed by centrifugation at 15,000× g for 10 min at 4 °C. Then, the tissue extract was incubated with McIlvaine buffer containing 0.2 M of Na_2_HPO_4_ (Sigma-Aldrich, Hamburg, Germany), 0.1 M of citric acid (Sigma-Aldrich, Hamburg, Germany), 0.23 mM of dichlorophenolindophenol (DCIP) (Sigma-Aldrich, Hamburg, Germany), and 0.1 mM of N6-isopentenyl adenine (Sigma-Aldrich, Hamburg, Germany) for 16 h at 37 °C.

The reaction was stopped by adding 0.3 mL of 40% trichloroacetic acid (TCA) (ROTH, Karlsruhe, Germany) and 0.2 mL of 2% PAF solution (4-aminophenol (ROTH) in 6% TCA). The product concentration was measured spectrophotometrically at wavelengths ranging from 300 to 700 nm. To determine the CKX activity, the total protein content was measured in each sample based on the Bradford assay [30] using bovine serum albumin (BSA) (Sigma-Aldrich, Hamburg, Germany) as a standard. The experiment was carried out for three to eight biological replicates, and two technical replicates were measured per sample.

### 2.4. Quantitative RT-PCR

The expression of the *HvCKX* and *HvIPT* genes was analyzed in seven DAP spikes and 10-day-old roots by quantitative real-time PCR. Total RNA was isolated using TRIzol reagent (Sigma-Aldrich, Hamburg, Germany) following the manufacturer’s instructions. First-strand cDNA was synthesized from 1 µg of RNA. DNaseI treatment and cDNA synthesis were performed using a Maxima H Minus First Strand cDNA Synthesis Kit (Thermo-Fisher Scientific, Waltham, MA, USA), according to the manufacturer’s protocol.

Primer pairs were designed for *HvCKX1* (HORVU3Hr1G019850), *HvCKX3* (HORVU1Hr1G042360), *HvIPT1* (HORVU2Hr1G062320), *HvIPT2* (HORVU5Hr1G055220), *HvIPT3* (HORVU0Hr1G006770), *HvIPT4* (HORVU1Hr1G011480), *HvIPT5* (HORVU5Hr1G110100), *HvIPT7* (HORVU3Hr1G025950), *HvIPT10* (HORVU7Hr1G120070), and *Elongation factor 2* (EF2) (AK250137.1) (Figure S3), which was used as a reference gene [23] (see Table S1 for the primer sequences). qPCR was carried out in a 15-µL mixture containing 1× Hot FIREPol EvaGreen qPCR Mix (Solis BioDyne, Tartu, Estonia), each primer at 0.4 µM, and 1 µL of template cDNA. The following temperature profile was used: an initial denaturation step of 95 °C for 15 min; 50 cycles of amplification at 95 °C for 20 s, 15 s of annealing (see Table S1 for annealing temperatures), and 72 °C for 20 s; and a melting curve profile from 72 to 95 °C, with the temperature rising 1 °C at each step and continuous fluorescence measurements. The expression levels of the *IPT* and *CKX* genes were calculated from three technical replicates according to the standard curve methods using the EF2 gene as a normalizer. The analysis was performed for three biological replicates, and three technical replicates were used per sample.

### 2.5. Transcriptome Analysis by RNA-seq

RNA-seq analysis was performed for the seven DAP spikes and 10-day-old roots of mutant and control plants. Three biological replicates from each line were analyzed. Total RNA was isolated using TRIzol reagent following the manufacturer’s protocol. The RNA samples were treated with DNaseI (Thermo-Fisher Scientific, Waltham, MA, USA) to remove any residual genomic DNA. A total of 6 µg of RNA per sample was used for library preparation. The libraries were constructed and sequenced by BGI Tech Solution Co., Ltd. (Hong Kong), using the BGISEQ-500 platform. The clean reads were mapped to the reference genome of barley IBSCv2 (EnsemblPlants) using HISAT [31]. The gene mapping was performed using Bowtie2 [32], and the expression levels were calculated with RSEM tool (RNA-Seq by Expectation Maximization) [33]. Differentially expressed genes between the mutant and control plants were identified using the DEseq2 algorithm [34]. Functional classification of the differentially expressed genes was performed online using the Mercator sequence annotation tool [35]. The genes were classified according to the MapMan BIN ontology [36]

### 2.6. Analysis of Phenotypic traits

Four yield-related traits were assessed in T_1_ homozygous mutant lines and control plants: the number of spikes, number of grains, grain weight, and 1000-grain weight, which was calculated by multiplying the mean grain weight by 1000. The analysis was performed for three biological replicates. The root phenotype was analyzed in the T_2_ seedlings of homozygous mutant lines and control plants, with seven to 10 biological replicates. Prior to germination, the seeds were placed on wet filter paper in Petri dishes and incubated at 4 ºC for three days for stratification. Then, the seedlings were grown in jars filled with glass beads and Hoagland medium [37]. After 10 days of cultivation, the root system of each seedling was scanned with an Epson V750 Pro scanner at 1200 dpi resolution. The total root length; root surface, volume, and diameter; and number of root hairs were analyzed using WinRHIZO software (Regent Instruments, Inc., Quebec, Canada). After scanning, the roots were dried on Whatman 3-MM paper and weighed. Then, the roots were immediately frozen in liquid nitrogen and subjected to further analyses.

### 2.7. Statistical Analysis

Statistical analysis was performed via Statistica v13.3 software (StatSoft, Kraków, Poland). The normality of the tested samples was verified by the Shapiro–Wilk test. For groups that fulfilled the normality assumption, one-way ANOVA and post hoc tests were performed to determine significant differences between groups at *p* < 0.01 and *p* < 0.05 confidence levels. Otherwise, a nonparametric Kruskal–Wallis test and multiple comparisons of median ranks were performed.

## 3. Results

### 3.1. Generation of Targeted Mutations in the HvCKX1 and HvCKX3 Genes in Barley

Knockout mutations of the *HvCKX1* and *HvCKX3* genes were induced by an RNA-guided Cas9 nuclease. The target sequences of both genes were selected at the 5′ proximal region of the first exon to ensure that no other *CKX* genes would be affected by the sgRNA constructs (Figures S1 and S2). Additionally, the putative off-target sites were searched by screening the entire sequence of the barley genome using CasOT software. It has been observed in plants that the target sequences with three or more mismatches are not recognized by the Cas9–sgRNA complex. No target sequences with fewer than three mismatches were found (Tables S2 and S3). However, to exclude the potential effects of undetected off-targets on the experiment, we used null segregants instead of wild-type plants as controls. The null segregants were derived from transgenic T_0_ lines that expressed a functional Cas9–sgRNA complex, and nontransgenic T_1_ segregants with wild-type *HvCKX1* and *HvCKX3* genes were selected as control plants (CT). As a result of genetic transformation, 71 ckx1-sgRNA and 53 ckx3-sgRNA independent transgenic plants were generated (Table 1). All the transformants were screened for the presence of mutations in either the *CKX1* or *CKX3* gene by PCR/RE (restriction enzyme) analysis. In total, 47 ckx1 and 37 ckx3 mutant plants were obtained (Table 1).

**Figure 1:**
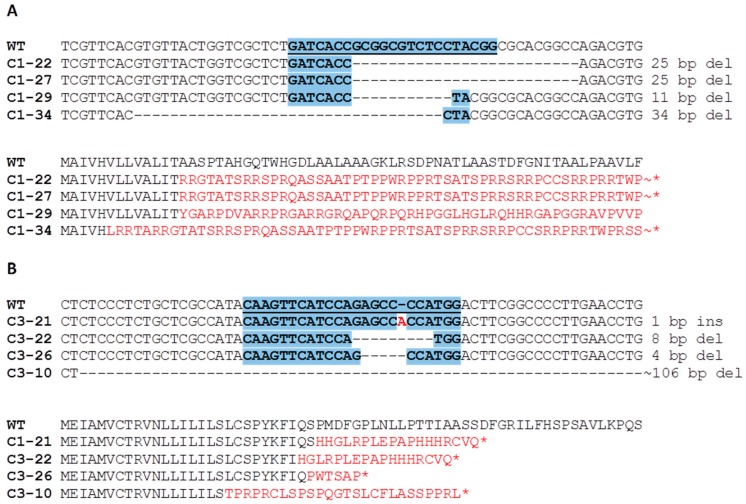
Types of mutations detected in the *HvCKX1* (**A**) and *HvCKX3* (**B**) genes of T_1_ lines and changes in the predicted amino acid sequences encoded by mutant alleles. Target sequences of the RNA-guided Cas9 nuclease are shaded in blue. Amino acid sequences resulting from the frameshift are marked in red.

Among the T_1_ progeny, 10 mutant plants were selected for segregation. Ten to 20 plants from each T_1_ line were screened to select transgene-free homozygous mutants. Four ckx1 mutant lines—C1-22, C1-27, C1-29, and C1-34—and four ckx3 mutant lines—C3-10, C3-21, C3-22 and C3-26—met the abovementioned criteria and were chosen for further analyses (Figure 1). The mutations induced in these lines led to loss of function as a result of either a frameshift in the open reading frame (ORF) or a frameshift and a premature stop codon. Interestingly, the deletion of the same 25-bp sequence occurred in mutants C1-22 and C1-27, despite both plants segregating from different transgenic events. Moreover, a large deletion of a 106-bp fragment occurred in mutant line C3-10, which is rather unusual when only a single DSB (double strand break) is generated.

### 3.2. CKX Activity in the Spikes and Roots

**Figure 2:**
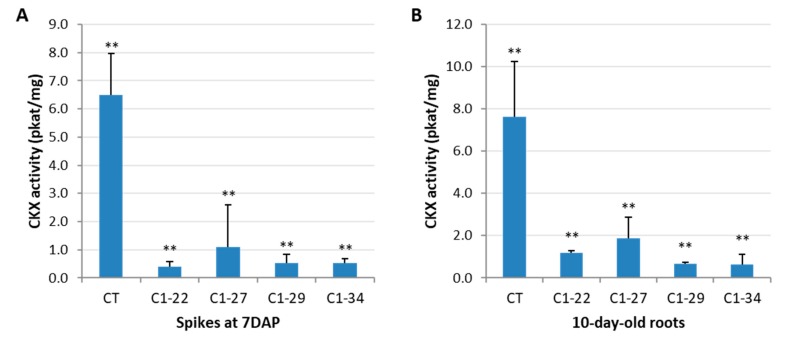
Enzymatic cytokinin oxidase/dehydrogenase enzymes (CKX) activity (pkat/mg) in seven days after pollination (DAP) spikes (**A**) and the 10-day-old roots (**B**) of ckx1 mutant lines. CT, control plants. The activity assay was carried out for three to eight biological replicates, and two technical replicates were measured per sample, which was significantly different from the control at ** *p* < 0.01.

**Figure 3:**
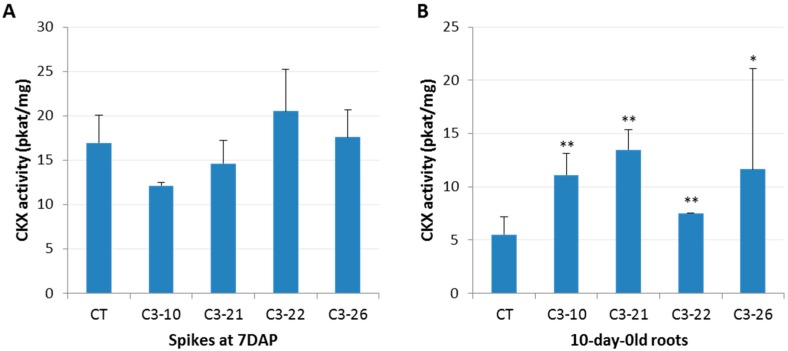
Enzymatic CKX activity (pkat/mg) in seven DAP spikes (**A**) and 10-day-old roots (**B**) of ckx3 mutant lines. CT, control plants. The activity assay was carried out for three to eight biological replicates, and two technical replicates were measured per sample significantly different from control at * *p* < 0.05, ** *p* < 0.01.

The activity of the CKX enzyme was estimated in the ckx1 and ckx3 mutant lines in developing spikes at seven DAP and in 10-day-old roots. A significant decrease in CKX activity ranging from 83% to 94% was observed in the seven DAP spikes (Figure 2A, Table S4) and from 75% to 90% in the roots of all the ckx1 mutant lines (Figure 2B, Table S4). In contrast, the CKX activity in the seven DAP spikes of the ckx3 lines was comparable to that of the control plants, except for line C3-10, in which only a slight decrease in activity was observed (Figure 3A, Table S5). Surprisingly, a significant increase in CKX activity ranging from 36% to almost 150% was observed in the roots of all the ckx3 lines compared to the control plants (Figure 3B, Table S5).

### 3.3. Expression of IPT Genes in the ckx1 and ckx3 Mutant Lines

**Figure 4:**
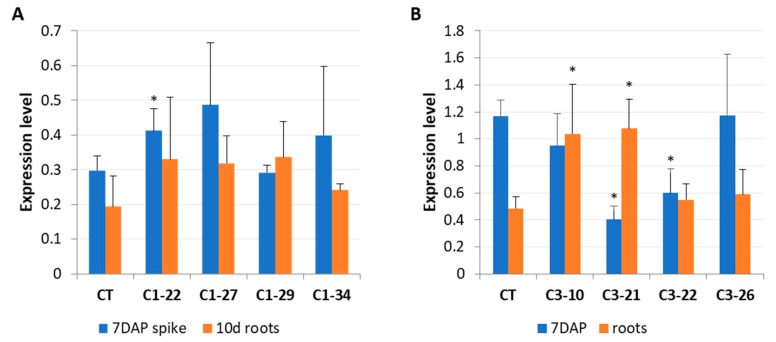
Expression levels of wild-type *HvCKX3* in ckx1 mutant lines (**A**) and wild-type *HvCKX1* in ckx3 mutant lines (**B**). The transcript accumulation was analyzed in seven DAP spikes and 10-day-old roots. CT, control plants; values are mean ± SD (Tables S6 and S7); significantly different from control at * *p* < 0.05. The expression levels were calculated from three technical replicates according to the standard curve methods using the *Elongation factor 2* gene as a normalizer. The analysis was performed for three biological replicates.

In previous reports [23,24], the expression of *HvCKX1* and *HvCKX3* was shown to be highest in developing seeds and roots. Therefore, we examined whether the expression of wild-type *HvCKX1* and *HvCKX3* was altered in the ckx3 and ckx1 mutants, respectively. No significant changes in *HvCKX3* expression were observed in the ckx1 mutant line, except for line C1-22, in which the tested gene showed increased expression (Figure 4A). In turn, a significant decrease in *HvCKX1* expression was observed in the seven DAP spikes of two ckx3 mutant lines: C3-21 and C3-22. Interestingly, the expression of *HvCKX1* was upregulated in the roots of lines C3-10 and C3-21 (Figure 4B). 

**Figure 5:**
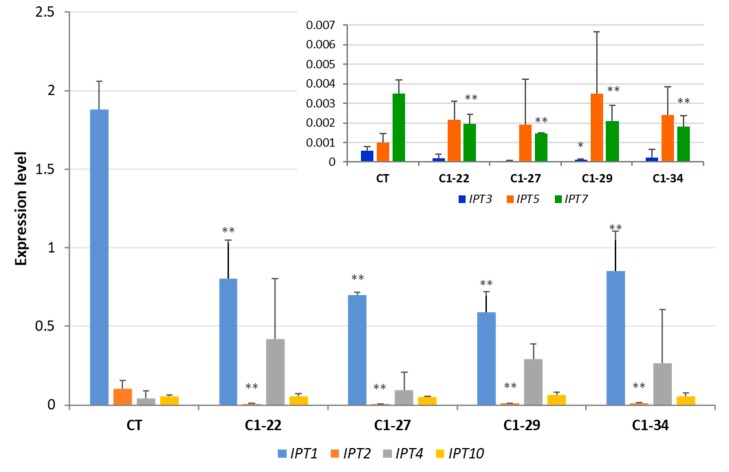
Expression levels of *HvIPT* genes in seven DAP spikes of ckx1 mutant lines; the expression of *HvIPT3*, *HvIPT5* and *HvIPT7* was shown on a separate graph because of lower expression level. CT, control plants; values are mean ± SD (Table S8) significantly different from the control at * *p* < 0.05, ** *p* < 0.01. The expression levels were calculated from three technical replicates according to the standard curve methods using the *Elongation factor 2* gene as a normalizer. The analysis was performed for three biological replicates.

**Figure 6:**
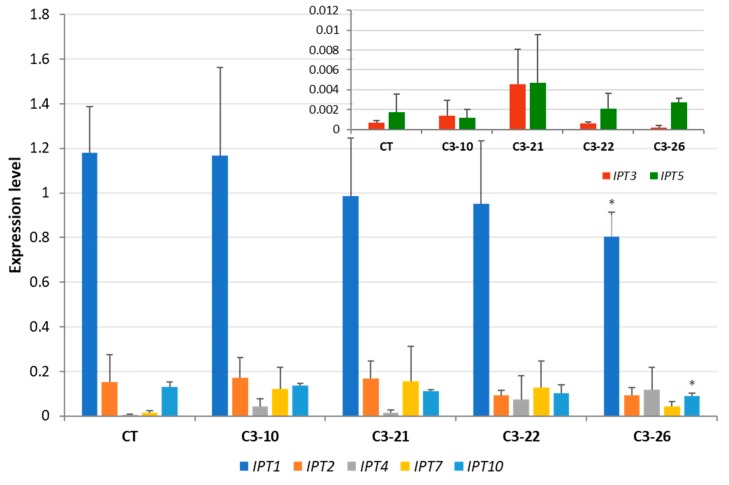
Expression of *HvIPT* genes in seven DAP spikes of ckx3 mutant lines; expression of *HvIPT3* and *HvIPT5* and was shown on a separate graph because of lower expression levels. CT, control plants; values are mean ± SD (Table S9) * significantly different from control at *p* < 0.05. The expression levels were calculated from three technical replicates according to the standard curve methods using the *Elongation factor 2* gene as a normalizer. The analysis was performed for three biological replicates.

To determine whether the expression of *IPT* genes was altered by the knockout of *CKX* genes, we examined the expression levels of seven known *HvIPT* genes in the roots of seedlings and in the developing seeds of the ckx1 and ckx3 mutant lines. The most highly expressed *IPT* gene family member was *IPT1* in the seven DAP spikes of control plants, while the expression levels of the *IPT2*, *IPT4*, and *IPT10* genes were more than 20 times lower. The expression of the remaining three gene family members, *IPT3*, *IPT5*, and *IPT7*, was slightly above the detection threshold of RT-qPCR (Figure 5). In the ckx1 mutant lines, a significant decrease in the expression of *IPT1*, *IPT2*, *IPT3*, and *IPT7* was observed, while the expression of the remaining genes was comparable to that of the control plants (Figure 5). The expression patterns of the *IPT* genes were more homogenous in the spikes of ckx3 mutant lines than in the other lines. An increase in *IPT4* and *IPT7* expression was observed in all lines compared to the control plants; however, the changes were not significant, and the expression of the remaining genes was similar to that of the control plants (Figure 6). 

**Figure 7:**
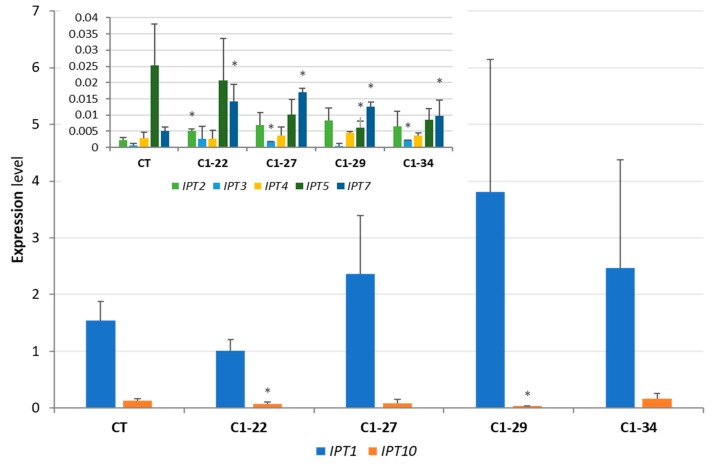
Expression levels of *HvIPT* genes in 10-day-old roots of ckx1 mutant lines; expression of *HvIPT2, HvIPT3, HvIPT4*, *HvIPT5*, and *HvIPT7* was shown on a separate graph because of their lower expression levels. CT, control plants; values are mean ± SD (Table S8) * significantly different from control at *p* < 0.05. The expression levels were calculated from three technical replicates according to the standard curve methods using the *Elongation factor 2* gene as a normalizer. The analysis was performed for three biological replicates.

**Figure 8:**
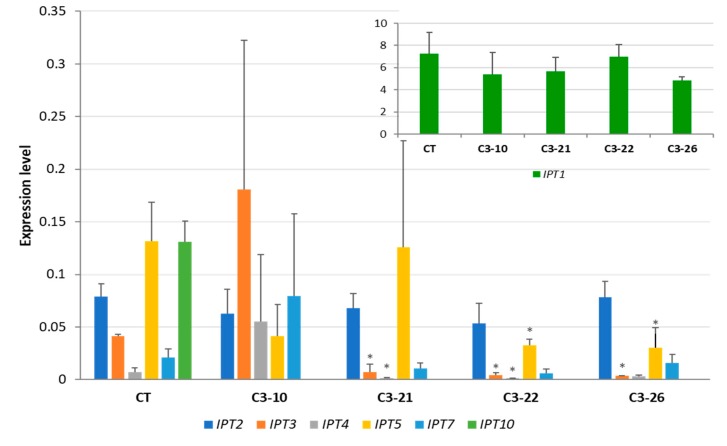
Expression levels of *HvIPT* genes in 10-day-old roots of ckx3 mutant lines; the expression of *HvIPT1* was shown on a separate graph because of the higher expression level. CT, control plants; values are mean ± SD (Table S9) * significantly different from control at *p* < 0.05. The expression levels were calculated from three technical replicates according to the standard curve methods using the *Elongation factor 2* gene as a normalizer. The analysis was performed for three biological replicates.

Different patterns of *IPT* expression were observed in 10-day-old roots. In the control plants, *IPT1* and *IPT10* were the most highly expressed genes; however, the expression level of *IPT10* was significantly lower than that of *IPT1*, while the transcript abundance of the remaining *IPT* genes was barely detectable. In the roots of mutant lines, no significant decrease in the expression level of *IPT* genes was observed in the ckx1 mutants, except for lines C1-22 and C1-29, which presented lower expressions of *IPT5* and *IPT10*. However, some of the other genes showed a slight increase in expression (Figure 7). The most apparent change in the ckx3 mutant lines was the decrease in *IPT10* expression to an undetectable level. Moreover, the expression of *IPT3*, *IPT4*, and *IPT5* decreased in all the lines except for C3-10 (Figure 8).

### 3.4. Transcriptome Analysis of the ckx1 and ckx3 Mutant Lines

As changes in CKX activity and in the expression of *IPT* genes have been observed in the mutant lines, we tested whether other biological processes were influenced by the knockout of the *CKX1* and *CKX3* genes. RNA-seq transcriptome analysis was performed on developing spikes and roots to identify differentially expressed genes (DEGs). Samples of three biological replicates of lines C1-22 and C3-21, whose CKX activity differed the most from control plants, were chosen for analysis. In all the samples, the number of both upregulated (log2 fold change ≥2, *p* value < 0.05) and downregulated (log2 fold change ≤−2, *p* value < 0.05) genes was approximately equal. In the C1-22 line, 366 upregulated and 406 downregulated genes were identified in seven DAP spikes, while the numbers of DEGs in the roots were 431 and 385, respectively. In the C3-21 line, 381 genes were upregulated in the spikes, and 341 were upregulated in the roots. Of the downregulated genes, 372 were identified in the spikes, and 292 were identified in the roots. To identify biological processes in which the DEGs were involved, we performed a functional annotation. The genes were classified according to the MapMan BIN ontology, which comprises 35 main biological categories. The full lists of annotated genes are presented in additional files, File S1 and File S2. The annotated DEG sequences represent a broad spectrum of biological processes (Tables S14 to S17). One characteristic of all the samples was the highest number of genes that could not be assigned (class 35) because of unknown function or no ontology in the databases. The second highest number of both upregulated and downregulated genes were classified as involving protein metabolism (class 29) and RNA processing (class 27). In all the samples, numerous differentially regulated genes were also related to stress (class 20), signaling (class 30), and hormone metabolism (class 17). One thing that may be important for seed and root development is the altered regulation of genes assigned to carbohydrate metabolism (classes 2 and 3), cell wall organization (class 10), and transport (class 34), which includes sugar transporters that were downregulated in the spikes. Moreover, in the roots of both mutant lines, we identified differentially regulated genes encoding proteins involved in the cytokinin signaling cascade (class 30.10). In the hormone metabolism class, 18 and 20 DEGs were identified in the seven DAP spikes of the ckx1 and ckx3 mutants, respectively, and the number of DEGs in the roots were seven and eight, respectively. The identified genes were assigned to different hormone subclasses, including those of auxins, cytokinins, gibberellins, jasmonic acid, salicylic acid, brassinosteroids, and ethylene.

We also manually searched among all the DEGs for genes that are directly involved in cytokinin metabolism, such as *CKX*, *IPT*, *ZOG*, and *GLU* (Table 2). A notable difference was observed between the analyzed tissues. Differentially expressed genes from the *CKX*, *IPT*, *ZOG*, and *GLU* families were found in the seven DAP spikes of both the ckx1 and ckx3 mutant lines, while in 10-day-old roots, only one member from each of the *CKX* and *LOG* families was found in the ckx3 mutant line. In the seven DAP spikes, differences in DEGs from the *CKX* family between the ckx1 and ckx3 lines were observed. In both mutant lines, the *CKX1* and *CKX3* genes indicated opposite regulation. In the C1-22 line, the wild-type *CKX3* gene was upregulated, while in the C3-21 line, the wild-type CKX1 gene showed downregulation. These results are consistent with those of the qPCR analysis in these lines. As expected, no changes in the expression of the mutant ckx1 and ckx3 genes were detected in the C1-22 and C3-21 lines, respectively. Interestingly, the expression of other CKX genes was also affected in both mutant lines. A downregulation of *CKX2.1* in the C3-21 line and *CKX4* and *CKX5* in both lines was observed. In turn, *CKX2.2* and *CKX9* in the C1-22 line were upregulated and downregulated, respectively, while the opposite regulation of these genes was observed in the C3-21 line. No apparent differences in the regulation of DEGs from the remaining families between the C1-22 and C3-21 lines were observed. Three out of four IPT genes showed the same type of regulation in both lines. Of the four putative zeatin *O*-glucosyltransferases, the orthologs of *TaZOG2* and *TaZOG3* were upregulated in both lines. A putative cis-zeatin *O*-glucosyltransferase, which is an ortholog of TacZOG1, was also upregulated in the C3-21 line. In contrast, a β-glucosidase gene was downregulated in both the C1–22 and C3-21 lines.

### 3.5. Analysis of Yield-Related Traits in the ckx1 and ckx3 Mutant Lines

**Figure 9:**
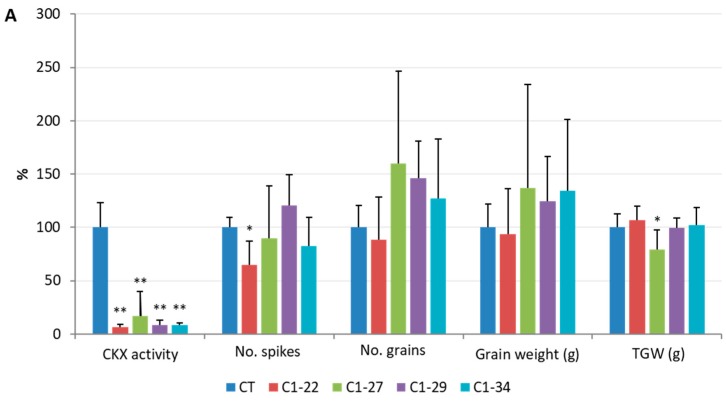

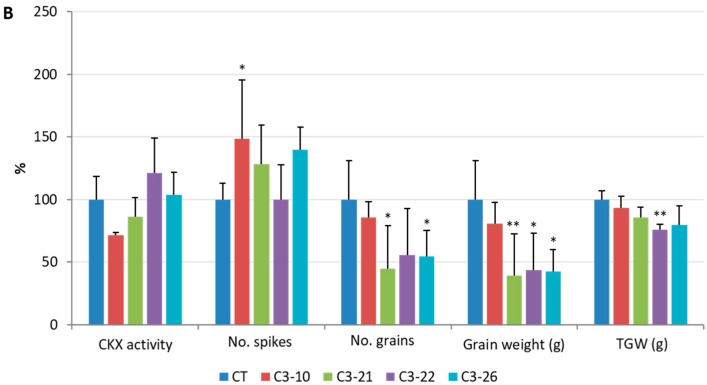
Comparison of enzymatic CKX activity and select yield parameters of seven DAP spikes of ckx1 (**A**) and ckx3 (**B**) mutant lines. The data are presented as the percentage of the values in control plants (CT), which were set to 100%, the actual values are shown in Tables S10 and S11. The yield parameters were measured for three biological replicates, which were significantly different from the control at * *p* < 0.05, ** *p* < 0.01.

To evaluate whether the knockout of the *HvCKX1* and *HvCKX3* genes and changes in CKX activity affected the phenotype of the mutant plants, we analyzed some of the yield-related parameters of the spikes and roots. The number of spikes, number of grains, mean weight of grains and thousand-grain weight (TGW) were measured in mature T_1_ plants, and the results were compared with the relative CKX activity (Figure 9). No significant changes were observed in the ckx1 mutant lines, except that line C1-22 had a lower number of spikes, and line C1-27 had a slightly lower TGW compared to the control plants. Lines C1-27, C1-29, and C1-34 showed a higher number of grains and grain weight; however, the changes were not statistically significant (Figure 9A). In turn, a significantly lower number of grains and grain weight were observed in three out of four ckx3 mutant lines: C3-21, C3-22, and C3-26 (Figure 9B). The number of spikes and TGW were comparable to those of the control plants, except for line C3-10, which had a higher spike number, and for line C3-22, which had a slightly lower TGW. 

**Figure 10:**
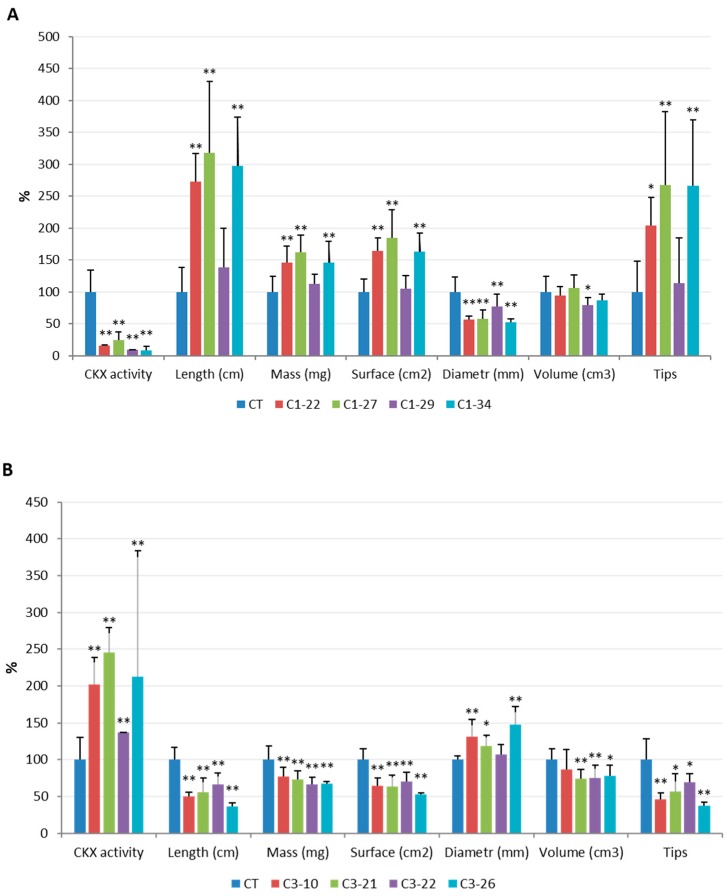
Comparison of enzymatic CKX activity and select morphological characteristics of 10-day-old roots from ckx1 (**A**) and ckx3 (**B**) mutant lines. The data are presented as the percentage of the values in control plants (CT), which were set to 100%, the actual values were shown in Tables S12 and S13. Root parameters were measured for seven to 10 biological replicates, which were significantly different from control at * *p* < 0.05, ** *p* < 0.01.

In contrast to the spikes, distinct visible changes between the control and mutant lines were observed in the root phenotypes of T_2_ plants and, more importantly, the effects were opposite in the ckx1 and ckx3 lines. Via high-resolution images of the scanned root systems of 10-day-old seedlings, basic morphological characteristics (such as total length; total root surface, diameter, and volume; and number of tips) were measured using WinRHIZO software. After being scanned, the fresh weight of the roots was also measured. The length values provided here indicate the total length of the entire root system, including both lateral roots and root hairs, and should not be mistaken for the average length of the primary roots. Again, the results were compared with the relative CKX activity in the roots (Figure 10). The total length of the roots of the ckx1 lines was significantly higher than that of the control plants, and the differences reached nearly 200% in lines C1-22, C1-27, and C1-34. Consequently, the fresh weight and total surface area of the roots were also higher in these lines than in control plants. Interestingly, the root diameter in all the ckx1 lines was markedly lower (approximately 50%) than that in the control plants, which may explain the fact that no notable changes in root volume were noticed. The approximate number of root hairs was also estimated and is represented by the tip parameter (Figure 10A). Compared to those of the control plants, the number of root hairs in lines C1-22, C1-27, and C1-34 was 100% to 150% higher and only slightly higher in line C1-29. As mentioned before, completely different phenotypic effects were observed in the roots of the ckx3 mutant lines. Both the total length and surface area as well as the fresh weight were significantly lower in the mutant lines than in the control plants; however, the differences in total length (from approximately 35% to 60%) were not as extensive as in the ckx1 lines (Figure 10B). The root diameter and volume were also more diversified in the ckx3 lines. The root diameter was higher in lines C3-10, C3-21, and C3-26; however, all the lines showed decreased root volume. The number of root hairs was also significantly lower in all the ckx3 lines than in the control plants, and the differences between the control and mutant lines ranged from 30% to 60%.

**Figure 11:**
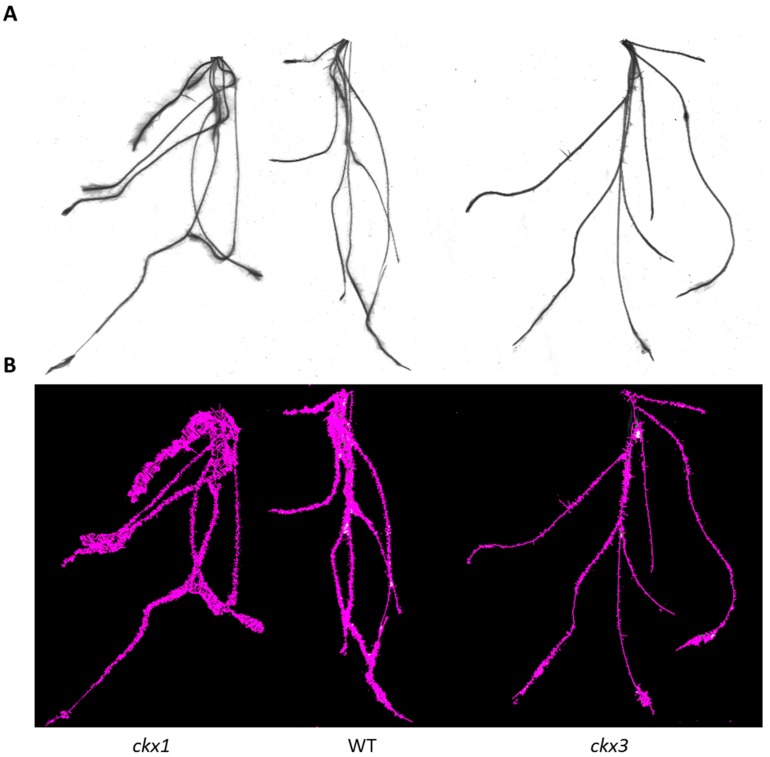
Examples of root phenotypes of the ckx1 mutant (left), a control plant (middle), and the ckx3 mutant (right) (**A**); differences in total surface area visualized in the images of scanned roots (**B**).

Differences in root phenotypes between the ckx1 and ckx3 mutants were visible in the scanned images, which also showed how the number of root hairs influenced the total surface area of the roots (Figure 11).

## 4. Discussion

In the past two decades, significant progress has been made in the study of cytokinin function and metabolism in plants. The simultaneous advancement in reverse genetics methods such as RNAi and genome editing has brought new possibilities for the direct regulation of genes involved in cytokinin metabolism. These achievements can be used either for improvement in crop yields or to elucidate the functions and interactions of individual elements of cytokinin metabolism. RNAi-based gene silencing has been used to study the function of *CKX* genes in many plant species [9]. The advantage of the RNAi approach is the possibility of either the constitutive or tissue-specific downregulation of a target gene. However, the incomplete and variable level of expression silencing, which depends on the transgene copy number and integration site [38], and which is sometimes not maintained through generations [39], are the major limitations of this method. Moreover, it should be taken into account that relatively long target sequences of approximately 300 to 800 bp are used in hpRNA (hairpin RNA) constructs [40], and RNAi is particularly amenable for off-target effects. These limitations can be overcome using modern genome-editing techniques, which offer the possibility of complete gene knockout by inducing precisely targeted mutations. The use of an RNA-guided Cas9 endonuclease adapted from the prokaryotic CRISPR/Cas9-based immune mechanism is a robust and highly efficient genome-editing system that has been successfully used in barley [3,4,5]. In this work, an RNA-guided Cas9 nuclease was used to introduce knockout mutations into either the *HvCKX1* or *HvCKX3* gene of the barley cultivar Golden Promise. Since the target sequence for sgRNA is an array only 20 bp upstream of the PAM motif (NGG) [41,42], it was chosen in the 5′ proximal, nonconservative coding region of both *CKX1* and *CKX3* to avoid off-targets in other *CKX* genes. In plants, the nucleolytic activity of Cas9 is completely abolished if at least one mismatch is present in the ‘seed’ region (a 12-bp fragment upstream of the PAM motif) of the protospacer sequence or if three or more mismatches occur in the entire protospacer sequence [43]. Based on the above criteria, we excluded the presence of potential off-targets by searching the complete sequence of the barley genome. However, to completely exclude the influence of potential off-targets, including those with noncanonical NAG and NGA PAMs [44] as well as other negative effects of genetic transformation, we chose null segregants with wild-type *HvCKX1* and *HvCKX3* from the progeny of T_0_ transformants as control samples in all the experiments. The frequency of induced mutations in T_0_ plants detected by PCR-RE analysis ranged from 66% to approximately 70% in the ckx1 and ckx3 lines, respectively. As we observed previously, the actual mutation frequency is usually higher, because the restriction enzymes used in the analysis can tolerate some of the SNP (single nucleotide polymorphism) mutations, giving false negative results [5]. Nevertheless, the efficiency of the RNA-guided Cas9 system was similar to that reported previously in barley and other cereals [4,45,46,47,48], and was sufficient for the selection of transgene-free homozygous mutant lines.

### 4.1. Cross-Regulation of Cytokinin Gene Family Members

The maintenance of cytokinin homeostasis is a complex process regulated by the balance of its biosynthesis, degradation, activation, and inactivation.

The biosynthesis and metabolism of cytokinin hormones are regulated by members of several multigene families, including isopentenyl transferases (*IPT*s) and LONELY GUY (*LOG*) for cytokinin biosynthesis and activation, cytokinin oxidases/dehydrogenases (*CKX*s) for irreversible degradation, zeatin *O*-glucosidases (*ZOG*s) for reversible inactivation, and β-glucosidases for reactivation [10,49]. The proper development of plant organs depends on the spatial and temporal coordinated regulation among these genes. In this study, we evaluated the effects of the knockout of the *HvCKX1* and *HvCKX3* genes and the resulting perturbations of CKX enzyme activity on the regulatory network of other cytokinin-related genes. In the first instance, we focused on the regulation of all the members of the *IPT* family. Cytokinin hormones are key regulators of seed development, and their production in developing seeds depends on the activity of several *IPT* gene family members (reviewed in [9]). *IPT* and *CKX* are the most important genes involved in cytokinin metabolic pathways, as the total content of bioactive cytokinins is directly related to their expression. In cereals, both *IPT* and *CKX* gene family members indicate tissue specificity and developmentally-dependent regulation [18,23,24,50,51]. In barley, the highest expression of the *HvCKX1* and *HvCKX3* genes was observed in young spikes between 0–14 DAP and in seedling roots [18,24]. The expression of *IPT* gene family members was detected in developing seeds between 0–14 DAP; however, at 14 DAP, the transcripts of most of the *IPT* gene family members were undetected. Therefore, the expression of *CKX1*, *CKX3*, and all the *IPT* gene family members was evaluated in spikes at seven DAP. In control plants, the highest expression was observed for *HvIPT1*, *HvIPT2*, and *HvIPT10*. The transcript abundance of the remaining four *IPT* gene family members was barely detectable by qRT-PCR. Similar expression activity during seed development was reported between the maize *ZmIPT1* and *ZmIPT2* and wheat *TaIPT2* orthologs [50,51,52]. In all the ckx1 mutant lines, the expression of both *HvIPT1* and *HvIPT2* was significantly lower than that in the control plants. This result suggests that a local increase in cytokinin content caused by the near-complete inactivation of CKX enzymes initiates a feedback mechanism that downregulates the expression of *IPT* gene family members. A similar effect was observed in maize seedlings after treatment with exogenous cytokinin, which resulted in the downregulation of *ZmIPT5* and *ZmIPT6* [53]. In turn, the CKX activity in the ckx3 mutant lines remained unchanged; thus, only a slight downregulation of *HvIPT1* was observed in line C3-26. However, RNA-seq analysis also showed the downregulation of this gene in the C3-21 line. In the roots of the control seedlings, *HvIPT1* had the highest expression; however, no significant change in its expression was observed in either the ckx1 or ckx3 mutant line. The second most expressed gene, *HvIPT10*, was downregulated in two ckx1 lines, but in all the ckx3 lines, its expression was not detected. This finding suggests that *HvIPT10* may be more important in CK biosynthesis in the roots, while *HvIPT1* and *HvIPT2* are the key genes of CK biosynthesis in developing seeds.

To identify other cytokinin-related genes that were differentially expressed in the mutant lines, the transcriptomes of the spikes and roots were sequenced by RNA-seq. The analysis confirmed the altered regulation of *IPT* genes in the spikes, i.e., the downregulation of *HvIPT1* and *HvIPT2*. Other genes involved in cytokinin metabolism and processing were also identified, including members of the *CKX*, *ZOG*, *LOG*, and *GLU* families. Analysis of the DEGs revealed that apart from cross-regulation between different members of cytokinin-related families, such as *CKX* versus *IPT* or *ZOG* versus *GLU*, internal regulation between members of the same gene family also exists. For example, five *CKX* genes that are normally weakly expressed in seven DAP spikes were identified among the DEGs. Moreover, some of these genes were inversely regulated in the ckx1 and ckx3 mutants; e.g., *HvCKX2.2* and *HvCKX9* were upregulated and downregulated in the ckx1 mutants, respectively, while the opposite regulation of these genes occurred in the ckx3 mutants. Similar internal regulation between *CKX* gene family members was observed during the development of different wheat organs. For instance, a strong correlation of expression between *TaCKX1*, *TaCKX2.1*, and *TaCKX2.2* was observed in consecutive stages of spike development between 0–14 DAP [54].

Active cytokinin forms can be released from their nucleotide precursors in a single-step reaction catalyzed by the cytokinin nucleoside 5′-monophosphate phosphoribohydrolase enzyme LONELY GUY (LOG) [49]. In rice, transcripts of the *LOG* gene were detected mainly in the shoot apical meristem (SAM), and a loss-of-function mutation causes the premature termination of generative meristems and a reduction in SAM size, resulting in small panicles with defective flowers [55]. Similar to *IPT* genes, *LOG*s are selectively regulated in response to changes in cytokinin content. Of the seven barley orthologs of rice *LOG*s identified among the DEGs, six were downregulated, and one was upregulated in the ckx3 mutant. Differences in the expression changes of *LOG*s were also observed in maize seedlings after treatment with exogenous cytokinin analogs. *LOG3* and *LOG4* were downregulated 72 h after treatment, while *LOG1* expression increased [53]. Apart from CKX-mediated degradation, glucosylation is another form of suppression of bioactive cytokinin. *O*-glucosylation is a reversible reaction that is catalyzed by zeatin *O*-glucosyltransferases and cis-zeatin *O*-glucosyltransferases [56,57], while in a reverse reaction, the glucosylated forms are hydrolyzed by β-glucosidase [58]. In both the ckx1 and ckx3 mutant lines, we observed an apparent coordinated regulation of *ZOG* and *GLU* genes. All the identified *ZOG* and *cZOG* genes were upregulated in the spikes with the simultaneous downregulation of *GLU*. A similar correlation between *ZOG* and *GLU* expression has been observed during seed development in wheat [50]. This process indicates that in the case of the knockout of *CKX* genes, excess cytokinin is inactivated in the glucosylation pathway. As shown in tobacco plants, such a reserve of inactive zeatin *O*-glucosides can be stored in the vacuole and, if needed, reactivated by β-glucosidase [59].

The above examples show that the genetic regulation of cytokinin metabolism and activation is a complex process that involves interactions between different gene families. However, at the molecular level, the mechanism of cytokinin signaling and crosstalk with other plant hormones is even more complex. In this study, we identified several differentially expressed genes that were classified into various subclasses of hormone metabolism and signaling, including those of auxins, gibberellins, brassinosteroids, jasmonic acid, and salicylic acid. The detailed analysis of each of these genes falls outside the scope of this research. However, it is known that cytokinin–auxin crosstalk plays a key role in cell fate specification and organ development; in roots, both hormones interact antagonistically, while in the shoot apical meristem, this interaction may be synergistic (reviewed in [60,61]). Some evidence has indicated that cytokinin-brassinosteroid crosstalk occurs both in plant development and in the abiotic stress response [62,63]. Salicylic acid (SA) and jasmonic acid (JA) are other plant hormones involved in the abiotic stress response [64,65]. There is little information about the direct interplay between cytokinins and both SA and JA in this process; however, some studies have confirmed their coregulation induced by biotic stress [66]. It is worth to note that aside from metabolism and signaling, hormone transport also plays a pivotal role in plant development. Different types of cytokinin that are synthesized in shoots and roots can move basipetally and acropetally through phloem and xylem, respectively [67]. Although it has become evident that cytokinins are transported, for a long time, little was known about the molecular background of this mechanism. In recent years, three types of cytokinin transporters have been identified: PUP (purine permeases) and ENT (equilibrative nucleoside transporters) for cytokinin uptake, and ABCG (G subfamily ATP-binding cassette) for cytokinin export [68]. Although we did not find any putative cytokinin transporters among the differentially expressed genes, this aspect should be further studied in barley.

### 4.2. Knockout of HvCKX1 and HvCKX3 Did Not Enhance Grain Yields but Affected the Root Phenotype

Since *HvCKX1* and *HvCKX3* are mostly expressed in developing seeds and in seedling roots, we evaluated the basic yield parameters as well as the root morphology of both mutant lines, and the results were compared with the CKX activity in those organs.

The final seed yield depends on the number of flowers, which is usually fixed in the early stages of reproductive organ development, and on seed size/weight, which is shaped later during seed development. Cytokinin hormones may be involved in both of these developmental processes, and their influence on seed yield has been well established in numerous studies [9]. There is also evidence that *CKX* genes may affect seed number and seed weight in both positive and negative manners. In naturally occurring rice mutants with a nonfunctional Osckx2 and wheat mutants with a nonfunctional *TaCKX6-D1*, an increased seed number and 1000-grain weight were observed, respectively [15,69]. A similar effect of increased seed number and/or seed weight was observed in rice, barley, and cotton after the downregulation of different *CKX* gene family members by RNAi [16,18,19,20,24,70]. In contrast, the ectopic expression of *CKX* genes in rice and Arabidopsis resulted in reduced seed or flower numbers [15,22]. It was believed that higher seed yields were stimulated by higher cytokinin contents, which resulted from the decreased activity of the CKX enzymes that catalyze the irreversible degradation of cytokinins. In the present study, there were differences in CKX activity detected in the spikes between the ckx1 and ckx3 mutants. The ckx1 lines showed a significant decrease in CKX activity compared with ckx3 lines, which did not differ from control plants. This difference may suggest that CKX enzymes indicate different specificities for the particular cytokinin hormone, and that *CKX3*-encoded enzymes had lower specificities for isopentenyladenine, which was used as a substrate in the activity assay. It is also possible that CKX activity in the ckx3 lines was at some range compensated by the upregulation of the *HvCKX9* gene. Despite these differences, no changes in yield parameters, including seed weight and number, were observed in either mutant line. The most likely explanation of this phenomenon is the activation of the homeostatic mechanism caused by increased cytokinin levels. As mentioned before, the knockout of *HvCKX1* and *HvCKX3* affected the regulation of other cytokinin-related genes. The most important effects were the downregulation of *HvIPT1* and *HvIPT2*, the main genes of cytokinin biosynthesis in developing seeds, and the upregulation of zeatin *O*-glucosyltransferase, which reversibly inactivates cytokinins with the simultaneous downregulation of β-glucosidase. Additionally, the direct cytokinin activation pathway was inhibited in some ranges, because several *LOG* genes were downregulated. The downregulation of *IPT* genes was less evident in the ckx3 mutants, but in turn, more *ZOG* genes were affected in the C3-21 line than in the C1-22 line. A similar homeostatic mechanism between *CKX* and *IPT* was confirmed in numerous studies in which cytokinin levels were elevated developmentally, by exogenous applications of cytokinin or by the overexpression of *IPT* genes [50,52,71,72,73].

Grain weight can also be positively influenced by cytokinin hormones via the upregulation of genes encoding cell wall invertase and hexose transporters during the grain-filling stage [74,75]. Both proteins are essential for the mobilization and flow of nutrients to storage organs [76]. We did not observe an altered regulation of cell wall invertase, although several genes classified as coding for hexose transporters were negatively regulated. Our results are in agreement with the findings of another group who studied the effect of *HvCKX1* knockout in barley [6]. Despite the higher seed number in the mutant lines, the average thousand-grain weight was 20% lower than that in the control plants. The authors suggest that other mechanisms, including the regulation of cell division, nutrient transport, and cell wall composition, may also be negatively affected by elevated levels of cytokinin. Interestingly, in our previous study, RNAi-based silencing of the *HvCKX1* gene resulted in a relatively high seed number and thousand-grain weight in the transgenic plants with decreased expression of this gene [18], and this effect was retained to the T_4_ generation [24]. This contradictory result may stem from two different reasons. First, in RNAi-based posttranscriptional gene silencing, the expression of the target gene is never suppressed by 100%, and the silencing level may vary during the life of a plant. The lowest transcript accumulation of *HvCKX1* observed in the T_1_ lines was 50% of that in the control plants. We hypothesize that this silencing level was not sufficient to induce a homeostatic response to the extent observed in the ckx1 and ckx3 knockout mutants from the present study. Second, the probability of a potential off-target effect is relatively high in the case of RNAi, and with the 413-bp fragment of *HvCKX1* used in the hpRNA cassette, it cannot be excluded that other *CKX* genes were targeted by complementary siRNA.

Auxin and cytokinin hormones play pivotal roles in root morphogenesis and development, and the antagonistic mechanism of their interaction in these organs is well known [49,77]. In contrast to auxin, cytokinin negatively influences root growth, and cytokinin-deficient plants produce relatively large root systems [23,78], while excess cytokinin inhibits root growth [79]. Our results are inconsistent with this model. Despite the suppression of CKX activity in all the ckx1 mutant lines, which is supposed to increase the cytokinin level, we observed increased root productivity, which manifested as increased root length, total surface area, and weight, as well as increased numbers of root hairs. In turn, in all the ckx3 mutant lines, the CKX activity was significantly higher compared to that in the control plants, which was probably caused by the upregulation of the *HvCKX1* gene. Despite the supposed decrease in cytokinin levels, all the productivity parameters of the root system were lower than those in the control plants. Interestingly, a positive effect of suppressed CKX activity on root growth was noticed in barley plants with the silenced expression of *HvCKX1* [18]. Apart from the relatively low expression of *HvIPT10* in the roots of both mutant lines, we did not observe changes in the regulation of other cytokinin metabolism genes, which might explain these contradictory results. However, it cannot be excluded that cytokinin signaling in the roots was affected in the ckx mutants. The core steps of cytokinin signaling have been well characterized in Arabidopsis. In short, after the binding of active cytokinin to histidine kinase receptor (AHK), a phosphorelay cascade is initiated in which the phosphoryl group is transferred via histidine phosphotransfer proteins (AHP) to Arabidopsis response regulators (ARRs). B-type *ARR*s act as positive regulators of cytokinin signaling, while A-type *ARR*s repress signaling via a negative feedback loop [11]. We observed different regulatory activities of a putative homolog of a phosphotransfer protein (AHP4), which was downregulated in the ckx1 mutant and upregulated in the ckx3 mutant. It has been indicated that the knockout of genes encoding AHP proteins leads to reduced cytokinin sensitivity [80]. Moreover, the upregulation of type-A *ARR*, which is a negative response regulator, was observed in the ckx1 mutant. It is possible that disturbances in cytokinin signaling may lead to partial insensitivity to cytokinin in the roots. However, such speculation needs to be supported by transcriptome analyses performed at different stages of root development and combined with cytokinin profiling. Therefore, additional studies are necessary to explore this aspect of cytokinin regulation in barley.

## 5. Conclusions

Cytokinins play a role in many aspects of plant development, and their positive impact on seed yield has been well documented. In the past decade, many efforts have been made to exploit endogenous cytokinins for yield improvement. The application of modern genome-editing methods brings new possibilities for crop improvement by manipulating cytokinin metabolism at the genetic level. In the present study, we used an RNA-guided Cas9 nuclease to produce mutant barley plants with inactivated an *HvCKX1* or *HvCKX3* gene, which are negative regulators of cytokinin content. Knockout of these genes and the supposed local increase in cytokinin levels in the spikes and roots activated the homeostatic response, which affected the regulation of other cytokinin-related genes. The suppressed CKX activity in the spikes was balanced by the reduced expression of *IPT* genes, which are involved in cytokinin biosynthesis, and the increased expression of *ZOG* genes, which are involved in cytokinin inactivation. These changes prevented the expected effect of increased grain yields. In turn, the unexpected changes in the root phenotype of both mutant lines were most likely caused by perturbations in cytokinin signaling. Our results demonstrate that the regulation of cytokinin activity is a complex mechanism, and that changes in only one element may have unexpected consequences. The evident cross-regulation of cytokinin-related genes should be taken into account in future efforts. To achieve the desired result of increased plant productivity, the homeostatic mechanism of cytokinin regulation must be effectively broken. For this purpose, it may be necessary to abolish the activity of other *CKX* gene family members and suppress cytokinin degradation even more, as well as abolish the activity of *ZOG* genes to prevent cytokinin inactivation. Such efforts would appear to be challenging. However, they are possible with novel genome-editing tools that allow for the simultaneous targeting of multiple genes by a single construct [5,45]. This approach could also be combined with the activation of expression of selected *IPT* genes by using an RNA-guided Cas9 system with catalytically inactive dCas9 fused to transcriptional activators.

## Figures and Tables

**Table 1:** Summary of the genetic transformation of barley and the PCR/RE screening of transformants for the presence of induced mutations in the *HvCKX1* and *HvCKX3* genes.

sgRNA Construct	Edited Gene	No. Explants	No. Transgenic Events (%)	No. Independent Transgenic Plants	No. PCR-RE-Detected Mutants
ckx1-sgRNA	*HvCKX1*	1143	137 (12)	71	47
ckx3-sgRNA	*HvCKX3*	931	76 (8)	53	37

**Table 2:** List of differentially expressed genes involved in cytokinin metabolism identified in the ckx1 and ckx3 mutant lines.

Gene	Identifier	Description	C1-22 (ckx1)	C3-21 (ckx3)
Spikes at Seven DAP			Change in Expression (Fold Change)	
CKX1	HORVU3Hr1G019850	Cytokinin oxidase/dehydrogenase	nd *	downregulation (−8.03)
CKX2.1	HORVU3Hr1G027460	Cytokinin oxidase/dehydrogenase	nd	downregulation (−2.83)
CKX2.2	HORVU3Hr1G027430	Cytokinin oxidase/dehydrogenase	upregulation (4.39)	downregulation (−5.29)
CKX3	HORVU1Hr1G042360	Cytokinin oxidase/dehydrogenase	upregulation (7.50)	nd
CKX4	HORVU3Hr1G105360	Cytokinin oxidase/dehydrogenase	downregulation (−5.00)	downregulation (−5.87)
CKX5	HORVU3Hr1G075920	Cytokinin oxidase/dehydrogenase	downregulation (−4.70)	downregulation (−2.57)
CKX9	HORVU1Hr1G057860	Cytokinin oxidase/dehydrogenase	downregulation (−6.70)	upregulation (4.91)
IPT1	HORVU2Hr1G062320	tRNA isopentenyltransferase	downregulation (−4.16)	downregulation (−5.58)
IPT2	HORVU5Hr1G055220	Adenylate isopentenyltransferase	downregulation (−4.17)	nd
IPT4	HORVU1Hr1G011480	Adenylate isopentenyltransferase	upregulation (5.32)	upregulation (3.60)
IPT10	HORVU7Hr1G120070	tRNA isopentenyltransferase	downregulation (−5.88)	downregulation (−4.81)
cZOG1	HORVU2Hr1G096890	Putative cis-zeatin O-glucosyltransferase (ortholog of TacZOG1)	nd	upregulation (4.23)
ZOG2	HORVU2Hr1G004720	Putative zeatin O-glucosyltransferase (ortholog of TaZOG2)	upregulation (4.70)	upregulation (5.64)
ZOG3	HORVU7Hr1G038510	Putative zeatin O-glucosyltransferase (ortholog of TaZOG3)	upregulation (7.19)	upregulation (2.69)
ZOG3	HORVU2Hr1G075920	Putative zeatin O-glucosyltransferase (ortholog of TaZOG3)	nd	upregulation (3.73)
LOG	HORVU3Hr1G050770	Putative cytokinin riboside 5’-monophosphate phosphoribohydrolase (ortholog of LOC_Os01g40630.1)	nd	downregulation (−4.46)
LOG	HORVU3Hr1G066810	Putative cytokinin riboside 5’-monophosphate phosphoribohydrolase (ortholog of LOC_Os01g51210.1)	downregulation (−4.00)	nd
LOG	HORVU1Hr1G079570	Putative cytokinin riboside 5’-monophosphate phosphoribohydrolase (ortholog of LOC_Os05g46360.1)	downregulation (−6.04)	downregulation (−7.07)
LOG	HORVU4Hr1G005660	Putative cytokinin riboside 5’-monophosphate phosphoribohydrolase (ortholog of LOC_Os03g49050.1)	downregulation (−6.23)	nd
LOG	HORVU1Hr1G094290	Putative cytokinin riboside 5’-monophosphate phosphoribohydrolase (ortholog of LOC_Os05g51390.1)	downregulation (−7.02)	downregulation (−6.32)
LOG	HORVU5Hr1G124750	Putative cytokinin riboside 5’-monophosphate phosphoribohydrolase (ortholog of LOC_Os03g64070.1)	nd	upregulation (6.77)
GLU	HORVU2Hr1G023590	Β-glucosidase	downregulation (−8.24)	downregulation (−7.67)
10-day-old roots			change in expression (fold change)	
CKX1	HORVU3Hr1G019850	Cytokinin oxidase/dehydrogenase	nd	upregulation (0.53)
LOG	HORVU2Hr1G089620	Putative cytokinin riboside 5’-monophosphate phosphoribohydrolase (ortholog of LOC_Os04g43840.1)	nd	downregulation (−3.41)

* not detected.

## Supplementary Materials

Supplementary materials are available at https://www.mdpi.com/2073-4409/8/8/782/s1. **Figure S1** Schematic structure of *HvCKX1* gene and target sequence position. **Figure S2** Schematic structure of *HvCKX3* gene and target sequence position. **Figure S3** Melting curves and reaction efficiencies for primer pairs of *HvIPT* (A–G), *HvCKX1* (H), *HvCKX3* (I) and *EF2* (J) genes. **Table S1** List of PCR primers used in this study. **Table S2** List of potential off-target sites in the barley genome searched for the target sequence located in the *HvCKX1* gene. Mm. type—type of the mismatches, Mm. all—number of all mismatches. **Table S3** List of potential off-target sites in the barley genome searched for the target sequence located in the *HvCKX3* gene. Mm. type—type of the mismatches, Mm. all—number of all mismatches. **Table S4** Enzymatic CKX activity in 7 DAP spikes and 10-day-old roots of ckx1 mutant lines. **Table S5** Enzymatic CKX activity in 7 DAP spikes and 10-day-old roots of ckx3 mutant lines. **Table S6** Expression level of wild-type *HvCKX3* in ckx1 mutant lines. **Table S7** Expression level of wild-type *HvCKX1* in ckx3 mutant lines. **Table S8** Expression level of *HvIPT* genes in 7 DAP spikes and 10-day-old roots of ckx1 mutant lines. **Table S9** Expression level of *HvIPT* genes in 7 DAP spikes and 10-day-old roots of ckx3 mutant lines. **Table S10** Selected yield parameters of 7DAP spikes of ckx1 mutant lines. **Table S11** Selected yield parameters of 7DAP spikes of ckx3 mutant lines. **Table S12** Select morphological characteristics of 10-day-old roots of ckx1 mutant lines. **Table S13** Select morphological characteristics of 10-day-old roots of ckx3 mutant lines. **Table S14** Summary of the functional classification of differentially expressed genes identified in the 7 DAP spikes of *ckx1* mutant line C1-22. **Table S15** Summary of the functional classification of differentially expressed genes identified in the 10-day-old roots of *ckx1* mutant line C1-22. **Table S16** Summary of the functional classification of differentially expressed genes identified in the 7 DAP spikes of *ckx3* mutant line C3-21. **Table S17** Summary of the functional classification of differentially expressed genes identified in the 10-day-old roots of *ckx3* mutant line C3-21. **File S1** List of differentially expressed genes identified in the 7 DAP spikes and 10-day-old roots of ckx1 mutant line C1-22. Functional annotation was performed using the Mercator sequence annotation tool. **File S2** List of differentially expressed genes identified in the 7 DAP spikes and 10-day-old roots of ckx3 mutant line C3-21. Functional annotation was performed using the Mercator sequence annotation tool.
