# Supplementary material for: Knockout of the HvCKX1 or HvCKX3 Gene in Barley (Hordeum vulgare L.) by RNA-Guided Cas9 Nuclease Affects the Regulation of Cytokinin Metabolism and Root Morphology
Source: Cells. 2019 Jul 26;8(8):782. doi: 10.3390/cells8080782 (PMC6721474; doi:10.3390/cells8080782)
Supplement: Supplementary file 1 [file cells-08-00782-s001.zip › Supplementary tables.pdf]

Table S1 List of PCR primers used in this study

Gene	Primer forward	Primer reverse	Annealing temp. (°C)
HvCKX1	CGTACACCATCTCGTTCC	GTCGATCCACATCTGCTC	60
HvCKX3	CCAAGGGACATCTCTCTG	CTGTGCAGCTCTATCTCA	60
HvIPT1	GTTCCAGATTTGACTGTTG	CGACTCTTTCATTGACATAA	60
HvIPT2	CAGGAGCTATGGAGGAAG	CTTCCCACAGCTCTTCTC	60
HvIPT3	GTGTGGAAATGGAAAGGA	GTGGTAAAGCTGGATCTTG	61
HvIPT4	GAGGTGGATGAGGCTATG	GATCCTTCATTCTCATGCTC	61
HvIPT5	CGAGGTGTTCCTGAAGAG	GACCATGCTGCATTCTTG	60
HvIPT7	CAAGGTGGTTATTGTGATGG	GTGGACCTGGATCTTGTC	61
HvIPT10	CCTTCACCTTACCATACA	GGAAGTCATAATCCAGTTC	60
Hv_EF2	ATGGCAACATATGAAGATG	GGTCAAAGAAGTTCTCAC	60
hpt	ATGACGCACAATCCCACTATCCT	AGTTCGGTTTCAGGCAGGTCTT	68
ckx1-ps (target sequence)	CAGCTTTCACCGTAGCAGCA	AACGAGATGGTGTACGGCCA	66.5
ckx3-ps (target sequence)	TCTCTCTCTCGCCCTTTGCT	CATCTCCACCACAATGCCAT	67

Table S2 List of potential off-target sites in the barley genome searched for the target sequence located in the *HvCKX1* gene. Mm. type – type of the mismatches, Mm. all – number of all mismatches

# Location	Site	Mm.Type	PAM	Mm.All	Exon_info	Function
chr3H:58407963-58407986:+	GATCACCG_CGGCGTCTCCTA-CGGC	A00	A:NGG	0	on target (HORVU3Hr1G019850)	HvCKX1
chr4H:423905800-423905823:-	GAcCtCCt_CGGCGTCTCCTA-CGGG	A03	A:NGG	3	(HORVU4Hr1G051610)	Uncharacterized protein
chr1H:547771679-547771702:+	GtcCACCG_CGGCGTCTaCgA-CGGC	A22	A:NGG	4	(HORVU1Hr1G091230)	
chr3H:431184160-431184183:-	GtcCACCG_CGGCGTCTaCgA-CGGC	A22	A:NGG	4	(HORVU3Hr1G057440)	
chr3H:491895515-491895538:+	GATCtCCt_CGGCGTCagCTA-CGGA	A22	A:NGG	4	(HORVU3Hr1G064470)	
chr5H:598325234-598325257:-	GccaACtG_CGGCGTCTCCTA-CGGC	A04	A:NGG	4	(HORVU5Hr1G095440)	
chr7H:649319954-649319977:-	cAaCACCG_CGGaGTCTgCTA-CGGA	A22	A:NGG	4	(HORVU7Hr1G119190)	
chrUn:77796041-77796064:+	GccaACtG_CGGCGTCTCCTA-CGGC	A04	A:NGG	4	(HORVU0Hr1G013800)	

Table S3 List of potential off-target sites in the barley genome searched for the target sequence located in the *HvCKX3* gene. Mm. type – type of the mismatches, Mm. all – number of all mismatches

# Location	Site	Mm.Type	PAM	Mm.All	Exon_info	Function
chr1H:306445088-306445111:+	CAAGTTCA_TCCAGAGCCCCA-TGGA	A00	A:NGG	0	on target (HORVU1Hr1G042360)	HvCKX3
chr3H:650676876-650676899:+	CAAGgTCt_aCCAGAGCCCCg-AGGT	A22	A:NGG	4	(HORVU3Hr1G095710)	
chr3H:657359954-657359977:+	CAtGcTCA_cCaAGAGCCCCA-AGGC	A22	A:NGG	4	(HORVU3Hr1G097950)	
chr4H:31161598-31161621:-	CgAGaTCA_TCCAcAaCCCCA-CGGT	A22	A:NGG	4	(HORVU4Hr1G010410)	
chr5H:551830063-551830086:-	tAAGTgCA_TCtAGtGCCCCA-CGGT	A22	A:NGG	4	(HORVU5Hr1G076710)	
chr5H:624911182-624911205:+	CAtGgTCA_TCCAcAGCCaCA-GGGC	A22	A:NGG	4	(HORVU5Hr1G106720)	
chr5H:651947828-651947851:-	CgAGcTCA_TCCAGAGCCtCc-AGGT	A22	A:NGG	4	(HORVU5Hr1G118080)	
chrUn:84614824-84614847:-	CcAGcTCt_TCCAGAGCCaCA-AGGA	A13	A:NGG	4	(HORVU0Hr1G015650)	

Table S4 Enzymatic CKX activity in 7 DAP spikes and 10-day-old roots of ckx1 mutant lines.

	Spike at 7DAP		10-day-old roots	
**Line**	**Activity [pKat/mg]**	**SD**	**Activity [pKat/mg]**	**SD**
CT	6,49	1,48	7,64	2,61
C1-22	0,40	0,19	1,17	0,10
C1-27	1,11	1,48	1,88	1,00
C1-29	0,54	0,30	0,68	0,06
C1-34	0,53	0,14	0,65	0,46

CT – control plants; SD – standard deviation; values are mean ± SD (n = 3 to 8)

Table S5 Enzymatic CKX activity in 7 DAP spikes and 10-day-old roots of ckx3 mutant lines.

	Spike at 7DAP		10-day-old roots	
**Line**	**Activity [pKat/mg]**	**SD**	**Activity [pKat/mg]**	**SD**
CT	16,94	3,15	5,50	1,67
C3-10	12,14	0,35	11,10	2,02
C3-21	14,62	2,60	13,47	1,91
C3-22	20,53	4,71	7,52	0,02
C3-26	17,57	3,07	11,66	9,43

CT – control plants; SD – standard deviation; values are mean ± SD (n = 3 to 6)

Table S6 Expression level of wild-type *HvCKX3* in *ckx1* mutant lines.

	Spikes at 7DAP		10-day-old roots	
**Line**	**Expression**	**SD**	**Expression**	**SD**
CT	0,298	0,043	0,193	0,089
C1-22	0,413	0,063	0,330	0,178
C1-27	0,487	0,179	0,318	0,080
C1-29	0,292	0,021	0,336	0,102
C1-34	0,399	0,198	0,241	0,018

CT – control plants; SD – standard deviation; values are mean ± SD (n = 3); Expression values were normalized in relation to *Elongation factor 2* reference gene (AK250137.1).

Table S7 Expression level of wild-type *HvCKX1* in *ckx3* mutant lines.

	Spikes at 7DAP		10-day-old roots	
**Line**	**Expression**	**SD**	**Expression**	**SD**
CT	1,169	0,117	0,486	0,087
C3-10	0,948	0,237	1,037	0,368
C3-21	0,403	0,098	1,076	0,215
C3-22	0,598	0,181	0,548	0,120
C3-26	1,175	0,449	0,588	0,183

CT – control plants; SD – standard deviation; values are mean ± SD (n = 3); Expression values were normalized in relation to *Elongation factor 2* reference gene (AK250137.1).

Table S8 Expression level of *HvIPT* genes in 7 DAP spikes and 10-day-old roots of *ckx1* mutant lines.

	CT		C1-22		C1-27		C1-29		C1-34	
	Spikes at 7DAP									
Gene	Expression	SD	Expression	SD	Expression	SD	Expression	SD	Expression	SD
IPT1	1,87903	0,18081	0,80544	0,24339	0,69801	0,01881	0,59121	0,12970	0,85011	0,25722
IPT2	0,10476	0,05018	0,00947	0,00124	0,00620	0,00224	0,01299	0,00061	0,01089	0,00687
IPT3	0,00056	0,00024	0,00018	0,00023	0,00004	0,00003	0,00011	0,00006	0,00023	0,00042
IPT4	0,04248	0,04738	0,41781	0,38422	0,09569	0,11428	0,29192	0,09607	0,26617	0,33849
IPT5	0,00100	0,00046	0,00217	0,00093	0,00191	0,00233	0,00350	0,00316	0,00241	0,00143
IPT7	0,00350	0,00071	0,00195	0,00049	0,00145	0,00003	0,00207	0,00083	0,00179	0,00057
IPT10	0,05474	0,00746	0,05730	0,01439	0,05172	0,00280	0,06285	0,01709	0,05503	0,02169
	10-day-old roots									
IPT1	1,53721	0,34256	1,01228	0,19485	2,36232	1,03016	3,81464	2,33754	2,46394	1,91375
IPT2	0,00230	0,00082	0,00512	0,00068	0,00686	0,00396	0,00833	0,00382	0,00657	0,00455
IPT3	0,00056	0,00062	0,00273	0,00381	0,00177	0,00002	0,00048	0,00067	0,00218	0,00011
IPT4	0,00293	0,00174	0,00264	0,00269	0,00376	0,00246	0,00447	0,00038	0,00366	0,00077
IPT5	0,02527	0,01282	0,02057	0,01299	0,01011	0,00469	0,00604	0,00218	0,00848	0,00345
IPT7	0,00505	0,00135	0,01416	0,00534	0,01698	0,00127	0,01255	0,00152	0,00985	0,00471
IPT10	0,13539	0,02445	0,07090	0,03587	0,08613	0,06274	0,03373	0,00762	0,16337	0,09785

CT – control plants; SD – standard deviation; values are mean ± SD (n = 3); Expression values were normalized in relation to *Elongation factor 2* reference gene (AK250137.1).

Table S9 Expression level of *HvIPT* genes in 7 DAP spikes and 10-day-old roots of *ckx3* mutant lines.

	**CT**		**C3-10**		**C3-21**		**C3-22**		**C3-26**	
	**Spikes at 7DAP**									
**Gene**	**Expression**	**SD**	**Expression**	**SD**	**Expression**	**SD**	**Expression**	**SD**	**Expression**	**SD**
IPT1	1,1802	0,2057	1,1664	0,3963	0,9852	0,2614	0,9516	0,2806	0,8037	0,1103
IPT2	0,1517	0,1232	0,1701	0,0911	0,1672	0,0800	0,0916	0,0234	0,0919	0,0369
IPT3	0,0007	0,0002	0,0014	0,0015	0,0045	0,0035	0,0006	0,0001	0,0002	0,0002
IPT4	0,0046	0,0027	0,0427	0,0332	0,0140	0,0126	0,0743	0,1049	0,1180	0,1011
IPT5	0,0017	0,0019	0,0012	0,0009	0,0047	0,0049	0,0021	0,0016	0,0028	0,0004
IPT7	0,0135	0,0105	0,1216	0,0977	0,1562	0,1549	0,1273	0,1193	0,0431	0,0218
IPT10	0,1308	0,0201	0,1371	0,0091	0,1132	0,0052	0,1017	0,0367	0,0907	0,0109
	**10-day-old roots**									
IPT1	7,2658	1,9190	5,3972	1,9946	5,6890	1,2501	6,9698	1,0954	4,8639	0,2985
IPT2	0,0790	0,0124	0,0626	0,0235	0,0681	0,0138	0,0534	0,0194	0,0784	0,0151
IPT3	0,0414	0,0017	0,1810	0,1416	0,0074	0,0076	0,0040	0,0026	0,0036	0,0003
IPT4	0,0074	0,0037	0,0550	0,0639	0,0015	0,0004	0,0013	0,0003	0,0031	0,0011
IPT5	0,1314	0,0370	0,0411	0,0302	0,1257	0,1015	0,0326	0,0059	0,0302	0,0195
IPT7	0,0210	0,0084	0,0797	0,0777	0,0106	0,0054	0,0060	0,0042	0,0158	0,0080
IPT10	0,1308	0,0201	nd	-	nd	-	nd	-	nd	-

CT – control plants; SD – standard deviation; nd – not detected; values are mean ± SD (n = 3); Expression values were normalized in relation to *Elongation factor 2* reference gene (AK250137.1).

Table S10 Selected yield parameters of 7DAP spikes of *ckx1* mutant lines.

	No. spikes	SD	No. grains	SD	Grain weight [g]	SD	WTG [g]	SD
CT	12,00	1,15	88,71	18,28	3,26	0,71	36,84	4,73
C1-22	7,80	2,68	78,20	35,45	3,04	1,40	39,35	4,86
C1-27	10,75	5,91	141,75	77,12	4,47	3,15	29,18	6,76
C1-29	14,50	3,42	129,50	31,20	4,06	1,37	36,76	3,32
C1-34	9,89	3,26	112,89	49,25	4,38	2,19	37,59	6,06

CT – control plants; SD – standard deviation; WTG – weight of thousand grains; values are mean ± SD (n = 3)

Table S11 Selected yield parameters of 7DAP spikes of *ckx3* mutant lines.

Line	No. spikes	SD	No. grains	SD	Grain weight [g]	SD	WTG [g]	SD
CT	11,67	1,51	114,50	35,48	4,59	1,44	40,16	2,75
C3-10	17,33	5,51	98,33	14,19	3,70	0,78	37,44	3,73
C3-21	15,00	3,61	51,33	39,17	1,81	1,52	34,36	3,27
C3-22	11,67	3,21	64,00	42,51	1,99	1,36	30,55	1,74
C3-26	16,33	2,08	62,33	23,76	1,96	0,79	32,06	6,01

CT – control plants; SD – standard deviation; WTG – weight of thousand grains; values are mean ± SD (n = 3)

Table S12 Select morphological characteristics of 10-day-old roots of *ckx1* mutant lines.

Line	Length [mm]	SD	Mass [mg]	SD	Surface [cm2]	SD	Diameter [mm]	SD	Volume [cm3]	SD	No. tips	SD
CT	57,73	22,17	58,33	13,95	4,53	0,92	0,27	0,06	0,03	0,01	1000,11	479,63
C1-22	157,23	25,82	85,00	14,89	7,42	0,93	0,15	0,02	0,03	0,00	2037,71	444,26
C1-27	183,29	64,91	94,56	15,36	8,37	1,99	0,15	0,04	0,03	0,01	2672,22	1154,99
C1-29	79,88	35,10	65,57	8,81	4,75	0,94	0,21	0,05	0,02	0,00	1137,43	711,02
C1-34	171,48	44,08	84,88	19,86	7,39	1,32	0,14	0,02	0,03	0,00	2662,13	1038,30

CT – control plants; SD – standard deviation; values are mean ± SD (n = 7 to 10)

Table S13 Select morphological characteristics of 10-day-old roots of *ckx3* mutant lines.

Line	Length [mm]	SD	Mass [mg]	SD	Surface [cm2]	SD	Diameter [mm]	SD	Volume [cm3]	SD	No. tips	SD
CT	233,88	38,47	116,79	21,44	10,61	1,58	0,14	0,01	0,04	0,01	3672,32	1044,20
C3-10	116,15	14,93	89,57	15,27	6,87	1,13	0,19	0,03	0,03	0,01	1707,57	300,82
C3-21	129,67	45,18	85,00	13,52	6,75	1,59	0,17	0,02	0,03	0,00	2078,33	889,76
C3-22	155,53	35,90	77,50	11,83	7,44	1,32	0,15	0,02	0,03	0,01	2543,63	411,92
C3-26	84,69	12,34	78,40	4,04	5,60	0,19	0,21	0,04	0,03	0,01	1358,00	194,06

CT – control plants; SD – standard deviation; values are mean ± SD (n = 7 to 10)

Table S14 Summary of the functional classification of differentially expressed genes identified in the 7 DAP spikes of *ckx1* mutant line C1-22.

BIN class	Upregulation	Number of DEG	Downregulation	Number of DEG
	Description		Description	
1	PS; light reaction	3	PS; light reaction;	9
2	Major CHO metabolism; synthesis;	23	Major CHO metabolism; synthesis;	11
3	Minor CHO metabolism; degradation	16	Minor CHO metabolism; degradation	1
4	Glycolysis	2	Glycolysis; cytosolic branch	6
5	Fermentation; ADH	0	Fermentation; aldehyde dehydrogenase	1
6	Gluconeogenesis / glyoxylate cycle	0	Gluconeogenesis	1
7	OPP	0	OPP	2
8	TCA / org transformation; TCA; pyruvate DH.E1	1	TCA / org transformation; TCA; pyruvate DH.E1	12
9	Mitochondrial electron transport	3	Mitochondrial electron transport / ATP synthesis; NADH-DH; localization not clear	6
10	Cell wall; cellulose synthesis; degradation; modification	7	Cell wall; precursor synthesis; degradation; modification	7
11	Lipid metabolism;	6	Lipid metabolism; FA synthesis and FA elongation	14
12	N-metabolism	0	N-metabolism	1
13	Amino acid metabolism; synthesis; degradation	49	Amino acid metabolism; synthesis; degradation	12
15	Metal handling; regulation	1	Metal handling; regulation	1
16	Secondary metabolism;	14	Secondary metabolism; isoprenoids; carotenoids	4
17	Hormone metabolism; cytokinin synthesis-degradation; gibberellin synthesis-degradation;	31	Hormone metabolism; auxin; signal transduction, cytokinin synthesis/degradation; brassinosteroid; jasmonate synthesis/degradation; ethylene synthesis/degradation	19
18	Co-factor and vitamine metabolism	0	Co-factor and vitamine metabolism	1
19	Tetrapyrrole synthesis	0	Tetrapyrrole synthesis	2
20	Stress; abiotic; biotic	5	Stress	10
21	Redox	1	Redox; thioredoxin; ascorbate and glutathione	7
22	Polyamine metabolism	0	Polyamine metabolism; synthesis	1

23	Nucleotide metabolism	4	Nucleotide metabolism; phosphotransfer and pyrophosphatases	2
25	C1-metabolism	0	C1-metabolism	1
26	Miscellaneous; peroxidases; dynamin	10	Miscellaneous; oxygenases	12
27	RNA; processing	22	RNA; processing	39
28	DNA; synthesis; chromatin structure	10	DNA; synthesis; chromatin structure	9
29	Protein; aa activation; synthesis; ribosomal protein; 40S subunit S10	48	Protein; aa activation; synthesis; ribosomal protein;	84
30	Signaling; light; calcium	8	Signaling; in sugar and nutrient physiology; receptor kinases; calcium	18
31	Cell; organization	11	Cell; organization; cytoskeleton	17
33	Development; unspecified	2	Development; unspecified	7
34	Transport; p- and v-ATPases; potassium	10	Transport; sugars; metals; p- and v-ATPases	21
35	Not assigned; no ontology; unknown	57	Not assigned; no ontology	59

Table S15 Summary of the functional classification of differentially expressed genes identified in the 10-day-old roots of *ckx1* mutant line C1-22.

**BIN class**	**Upregulation**	**Number of DEG**	**Downregulation**	**Number of DEG**
	Description		Description	
1	PS; light reaction; photosystem I; photosystem II	3	PS; light reaction; PSII; LHC-II	0
2	Major CHO metabolism; synthesis; starch	1	Major CHO metabolism; degradation	6
3	Minor CHO metabolism; raffinose family;	2	Minor CHO metabolism;	3
4	Glycolysis; cytosolic branch	4	Glycolysis; cytosolic branch; pyruvate kinase	1
5	Fermentation; ADH	4	Fermentation; aldehyde dehydrogenase	0
6	Gluconeogenesis / glyoxylate cycle	0	Gluconeogenesis	0
7	OPP	0	OPP	0
8	TCA / org transformation; succinate dehydrogenase	1	TCA / org transformation; pyruvate DH.E3	1
9	Mitochondrial electron transport / ATP synthesis;	0	Mitochondrial electron transport / ATP synthesis;	1

	NADH-DH; complex I		NADH-DH; localization not clear	
10	Cell wall; synthesis; degradation	10	Cell wall; precursor synthesis	6
11	Lipid metabolism; FA synthesis; lipid degradation	9	Lipid metabolism; FA synthesis and FA elongation	10
12	N-metabolism	1	N-metabolism	1
13	Amino acid metabolism; synthesis	6	Amino acid metabolism; synthesis	5
15	Metal handling; regulation	0	Metal handling; regulation	0
16	Secondary metabolism; phenylpropanoids	14	Secondary metabolism; isoprenoids; carotenoids	6
17	Hormone metabolism; abscisic acid synthesis/degradation; jasmonate synthesis/degradation; auxin synthesis/degradation; salicylic acid synthesis degradation	22	Hormone metabolism; cytokinin synthesis / degradation; brassinosteroid	8
18	Co-factor and vitamine metabolism	1	Co-factor and vitamine metabolism	3
20	Stress; abiotic; biotic	26	Stress; biotic	19
21	Redox; heme	1	Redox; dismutases and catalases	2
22	Polyamine metabolism	0	Polyamine metabolism;	0
23	Nucleotide metabolism	3	Nucleotide metabolism;	2
25	C1-metabolism	0	C1-metabolism	0
26	Miscellaneous;	33	Miscellaneous;	13
27	RNA; processing; regulation of transcription; RNA binding	43	RNA; processing; splicing	55
28	DNA; synthesis; repair	12	DNA; synthesis; chromatin structure	17
29	Protein; degradation; folding; glycosylation	42	Protein; synthesis; initiation	45
30	Signaling; receptor kinases; calcium; G-proteins	26	Signaling; receptor kinases	26
31	Cell; organization; vesicle transport	10	Cell; organization	16
33	Development; unspecified	12	Development; unspecified	10
34	Transport	31	Transport; metabolite transporters at the mitochondrial membrane; ABC transporters	22
35	Not assigned; no ontology; unknown	113	Not assigned; no ontology; unknown	107

Table S16 Summary of the functional classification of differentially expressed genes identified in the 7 DAP spikes of *ckx3* mutant line C3-21.

**BIN class**	**Upregulation**	**Number of DEG**	**Downregulation**	**Number of DEG**
	Description		Description	
1	PS; light reaction; photosystem II; PSII polypeptide subunits	8	PS; light reaction; photosystem II; LHC-II	8
2	Major CHO metabolism; synthesis; starch	10	Major CHO metabolism; synthesis; starch	6
3	Minor CHO metabolism; others	5	Minor CHO metabolism; callose	6
4	Glycolysis; cytosolic branch; phosphoglycerate mutase	7	Glycolysis; cytosolic branch; phosphofructokinase	5
6	Gluconeogenesis / glyoxylate cycle	2	Gluconeogenesis	0
7	OPP	0	OPP	1
8	TCA / org transformation; IDH	7	TCA / org transformation; IDH	3
9	Mitochondrial electron transport / ATP synthesis;	2	Mitochondrial electron transport / ATP synthesis;	1
10	Cell wall; degradation	9	Cell wall; cellulose synthesis	7
11	Lipid metabolism;	13	Lipid metabolism; FA synthesis and FA elongation	7
13	Amino acid metabolism; synthesis	12	Amino acid metabolism; synthesis	30
15	Metal handling; regulation	1		0
16	Secondary metabolism	8	Secondary metabolism;	4
17	Hormone metabolism; auxin; induced-regulated-responsive-activated; cytokinin synthesis/degradation	23	Hormone metabolism; cytokinin synthesis / degradation; ethylene synthesis/degradation	15
18	Co-factor and vitamine metabolism	0	Co-factor and vitamine metabolism	3
19	Tetrapyrrole synthesis	0	Tetrapyrrole synthesis; glu-tRNA reductase	5
20	Stress;	26	Stress; abiotic	10
21	Redox; heme	1	Redox; thioredoxin	7
22	Polyamine metabolism; synthesis	1	Polyamine metabolism; synthesis	1
23	Nucleotide metabolism	3	Nucleotide metabolism;	2
24	Biodegradation of Xenobiotics	1		1

25	C1-metabolism	0	C1-metabolism	1
26	Miscellaneous; UDP glucosyl and glucoronyl transferases	22	Miscellaneous; UDP glucosyl and glucoronyl transferases	18
27	RNA; processing;	32	RNA; processing;	35
28	DNA; synthesis; chromatin structure	8	DNA; synthesis; chromatin structure	7
29	Protein; aa activation; synthesis; degradation	45	Protein; aa activation; synthesis	68
30	Signaling; receptor kinases;	6	Signaling; receptor kinases	15
31	Cell;	6	Cell; organization	19
33	Development; late embryogenesis	9	Development; unspecified	6
34	Transport; metal	15	Transport; sugars; metabolite transporters at the mitochondrial membrane; metal	22
35	Not assigned; no ontology; unknown	99	Not assigned; no ontology; unknown	14

Table S17 Summary of the functional classification of differentially expressed genes identified in the 10-day-old roots of *ckx3* mutant line C3-21.

**BIN class**	**Upregulation**	**Number of DEG**	**Downregulation**	**Number of DEG**
	Description		Description	
1	PS; light reaction; photosystem II; PSII polypeptide subunits	1	PS; light reaction; photosystem II; LHC-II	0
2	Major CHO metabolism; synthesis; starch	0	Major CHO metabolism; synthesis; starch	2
3	Minor CHO metabolism; others	1	Minor CHO metabolism; callose	2
4	Glycolysis; cytosolic branch; phosphoglycerate mutase	1	Glycolysis; cytosolic branch; phosphofructokinase	0
5	Fermentation; ADH	0	Fermentation; aldehyde dehydrogenase	0
6	Gluconeogenesis / glyoxylate cycle	0	Gluconeogenesis	0
7	OPP; oxidative PP	1		0
8	TCA / org transformation;	3	TCA / org transformation; IDH	1
9	Mitochondrial electron transport / ATP synthesis;	2	Mitochondrial electron transport / ATP synthesis;	1
10	Cell wall; degradation	3	Cell wall; degradation	2

11	Lipid metabolism; FA synthesis and elongation	9	Lipid metabolism; FA synthesis and FA elongation; degradation	2
12	N-metabolism	0	N-metabolism	1
13	Amino acid metabolism; synthesis	5	Amino acid metabolism; synthesis	2
15	Metal handling; regulation	0	Metal handling; regulation	2
16	Secondary metabolism; isoprenoids	9	Secondary metabolism; phenylpropanoids	10
17	Hormone metabolism; auxin; induced-regulated-responsive-activated; brassinosteroid signal transduction; jasmonate synthesis/degradation; salicylic acid synthesis/degradation	13	Hormone metabolism; auxin; synthesis / degradation;	4
18	Co-factor and vitamine metabolism	2	Co-factor and vitamine metabolism	6
19	Tetrapyrrole synthesis	0	Tetrapyrrole synthesis; glu-tRNA reductase	0
20	Stress; biotic; abiotic	16	Stress; biotic; abiotic	10
21	Redox	0	Redox; thioredoxin	3
22	Polyamine metabolism; synthesis	1	Polyamine metabolism; synthesis	0
23	Nucleotide metabolism	0	Nucleotide metabolism; degradation	4
25	C1-metabolism	1	C1-metabolism	0
26	Miscellaneous; UDP glucosyl and glucoronyl transferases	30	Miscellaneous; cytochrome P450	8
27	RNA; processing;	42	RNA; processing;	35
28	DNA; synthesis; chromatin structure	7	DNA; synthesis; chromatin structure	15
29	Protein; aa activation; synthesis	39	Protein; aa activation; synthesis	40
30	Signaling; in sugar and nutrient physiology	32	Signaling; in sugar and nutrient physiology; phosphorelay	22
31	Cell; organisation	15	Cell; organization, devision; cell cycle	9
33	Development; multitarget	3	Development; squamosa promoter binding like (SPL)	8
34	Transport; p- and v-ATPases	32	Transport; metal	14
35	Not assigned; no ontology; unknown	72	Not assigned; no ontology; unknown	85
