# Supplementary material for: Knockout of the HvCKX1 or HvCKX3 Gene in Barley (Hordeum vulgare L.) by RNA-Guided Cas9 Nuclease Affects the Regulation of Cytokinin Metabolism and Root Morphology
Source: Cells. 2019 Jul 26;8(8):782. doi: 10.3390/cells8080782 (PMC6721474; doi:10.3390/cells8080782)
Supplement: Supplementary file 1 [file cells-08-00782-s001.zip › Supplementary figures.pdf]

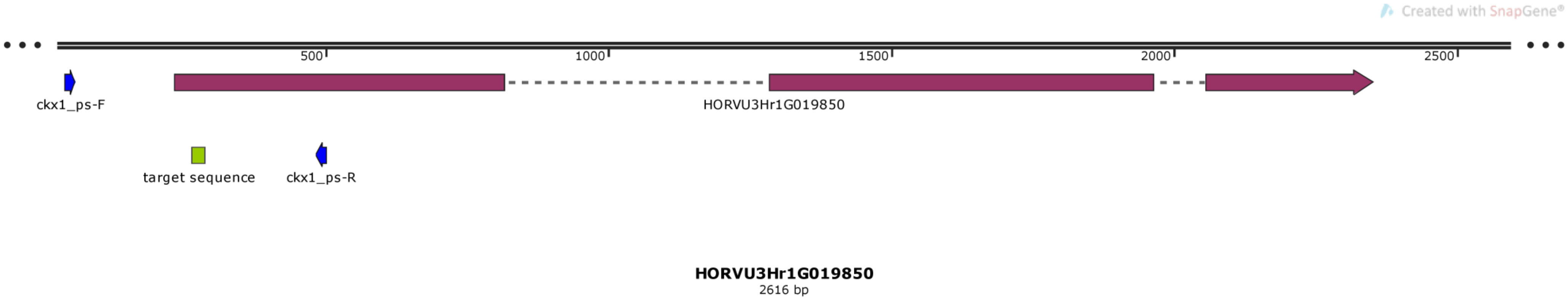

Fig. S1 Schematic structure of *HvCKX1* gene and target sequence position. Purple bars represent exons; position of target sequence is marked by green bar; position of ckx1-ps primers are marked by dark blue arrows.

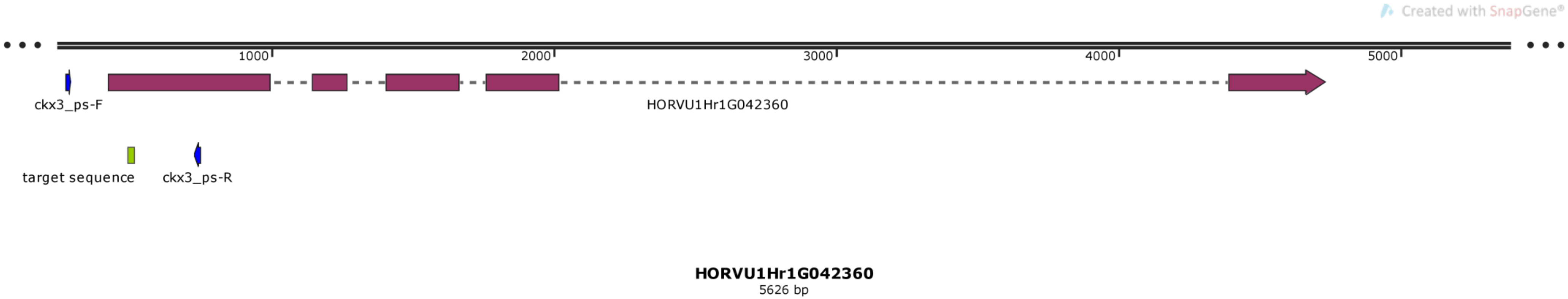

Fig. S2 Schematic structure of *HvCKX3* gene and target sequence position. Purple bars represent exons; position of target sequence is marked by green bar; position of ckx1-ps primers are marked by dark blue arrows.

Fig. S3

A) IPT1

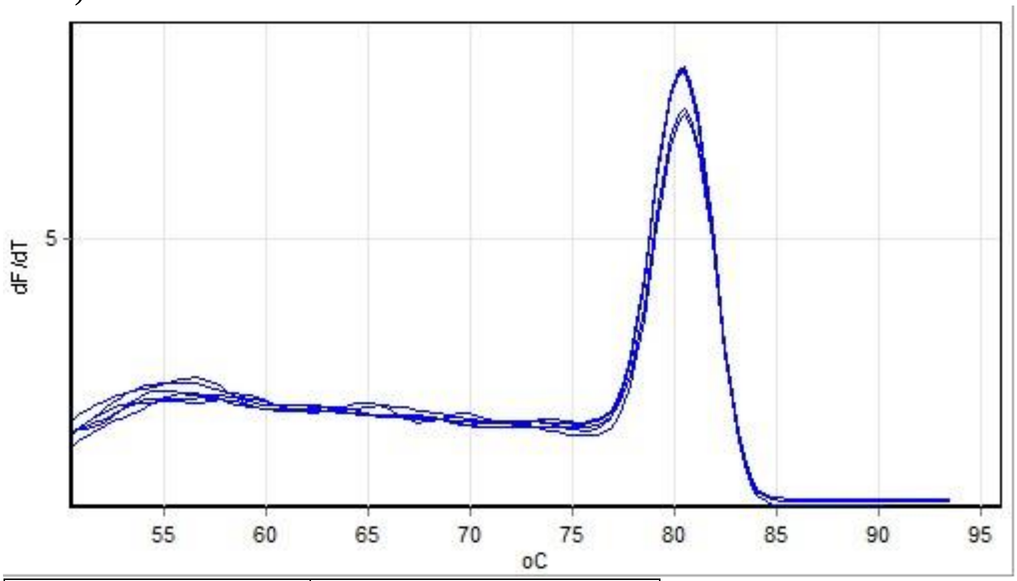

Reaction efficiency (*)	1,0053 (* = 10^(-1/m) - 1)

B) IPT2

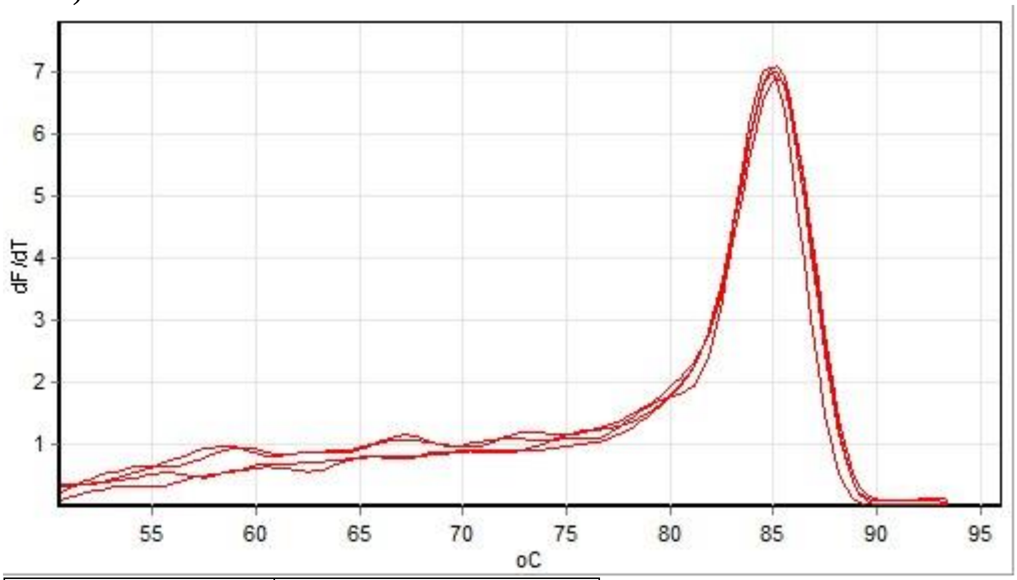

Reaction efficiency (*)	1,00773 (* = 10^(-1/m) - 1)

C) IPT3

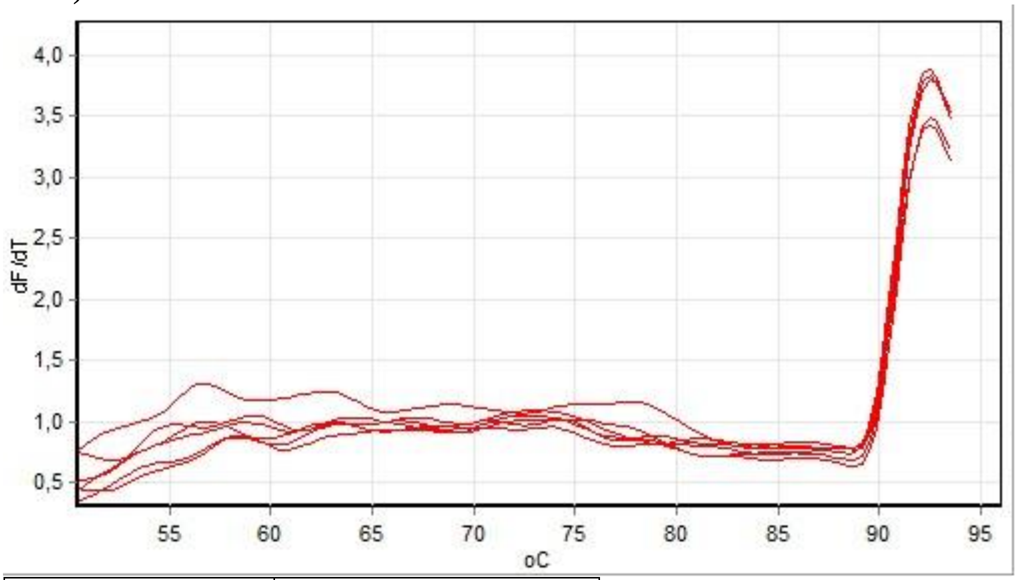

Reaction efficiency (*)	0,92709 (* = 10^(-1/m) - 1)

D) IPT4

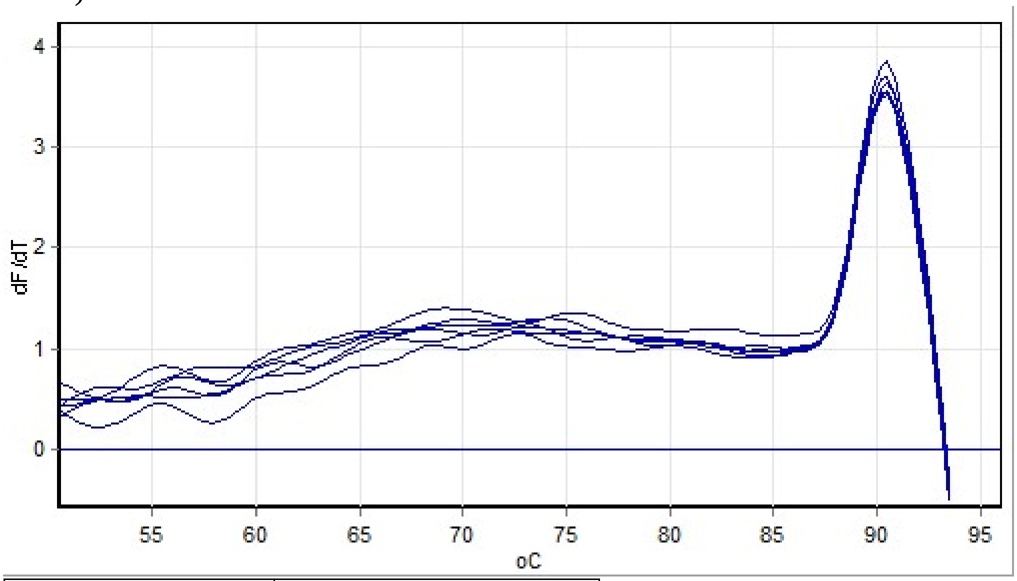

Reaction efficiency (*)	0,95254 (* = 10^(-1/m) - 1)

E) IPT5

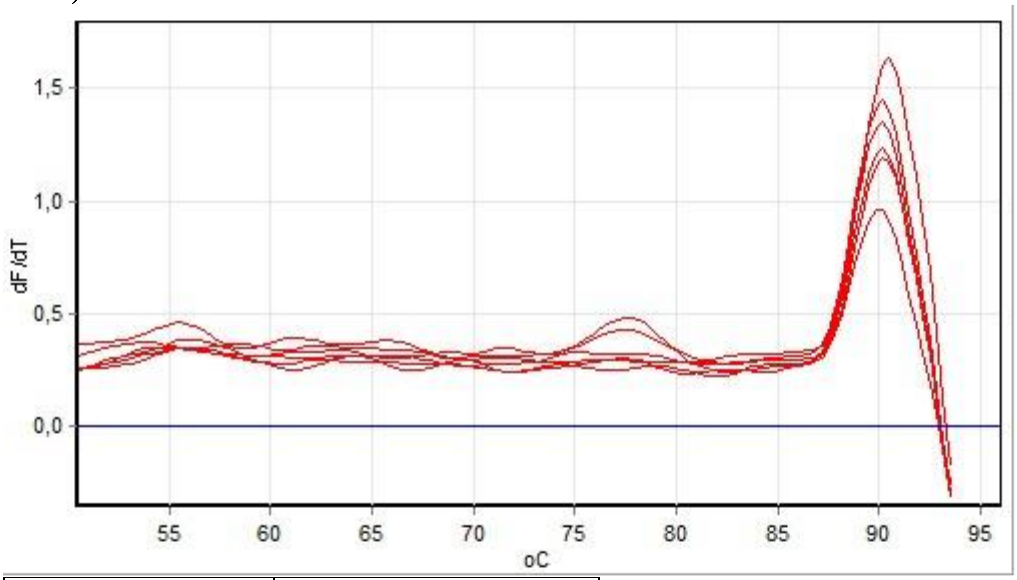

Reaction efficiency (*)	0,94357 (* = 10^(-1/m) - 1)

F) IPT7

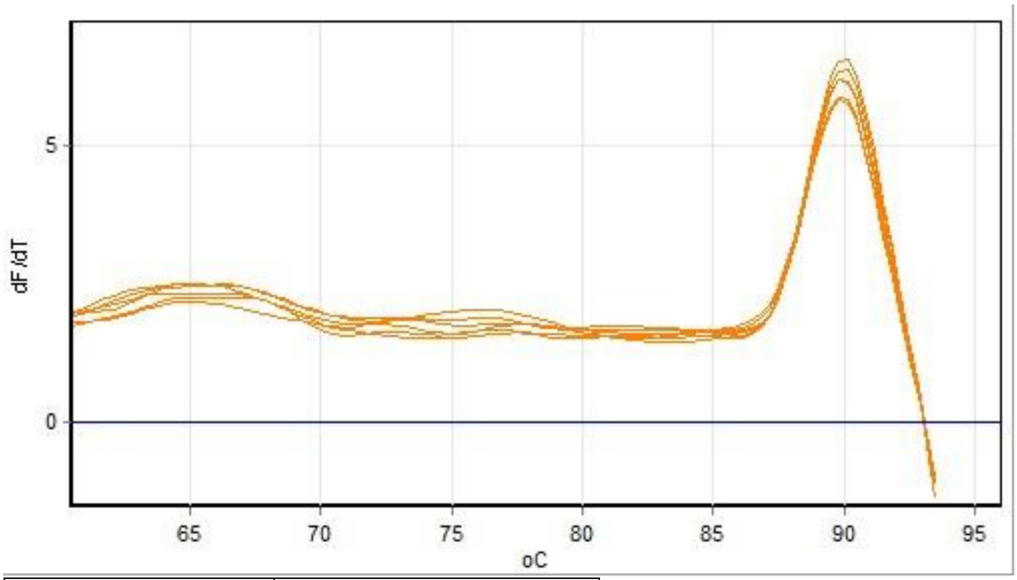

Reaction efficiency (*)	1,00093 (* = 10^(-1/m) - 1)

G) IPT10

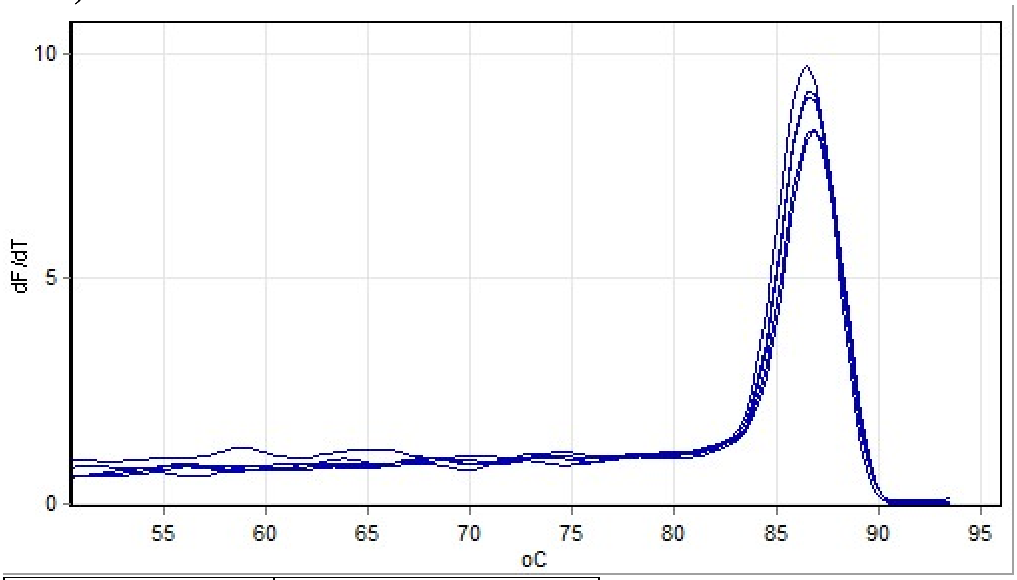

Reaction efficiency (*)	1,00448 (* = 10^(-1/m) - 1)

H) CKX1

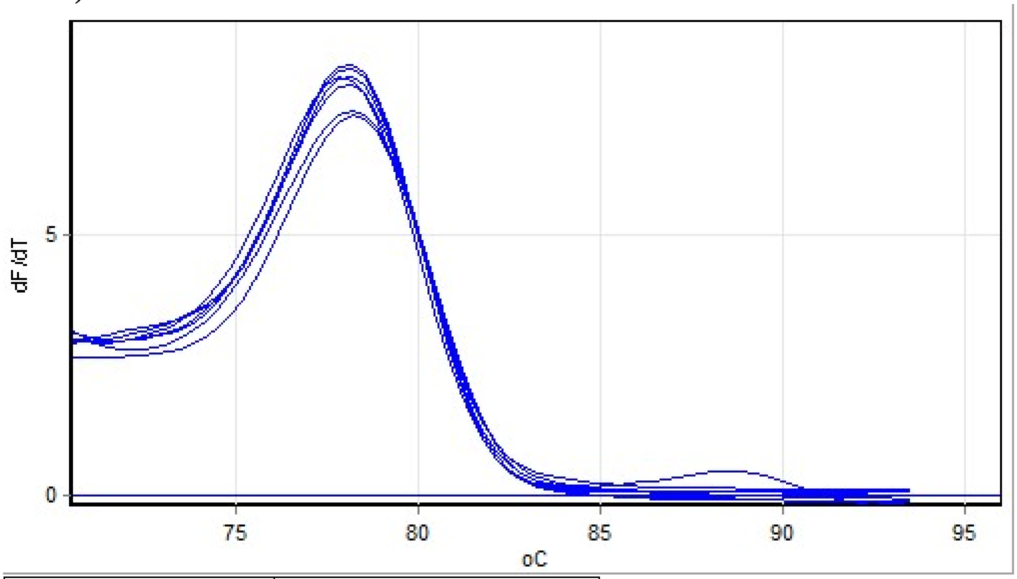

Reaction efficiency (*)	0,81026 (* = 10^(-1/m) - 1)

I) CKX3

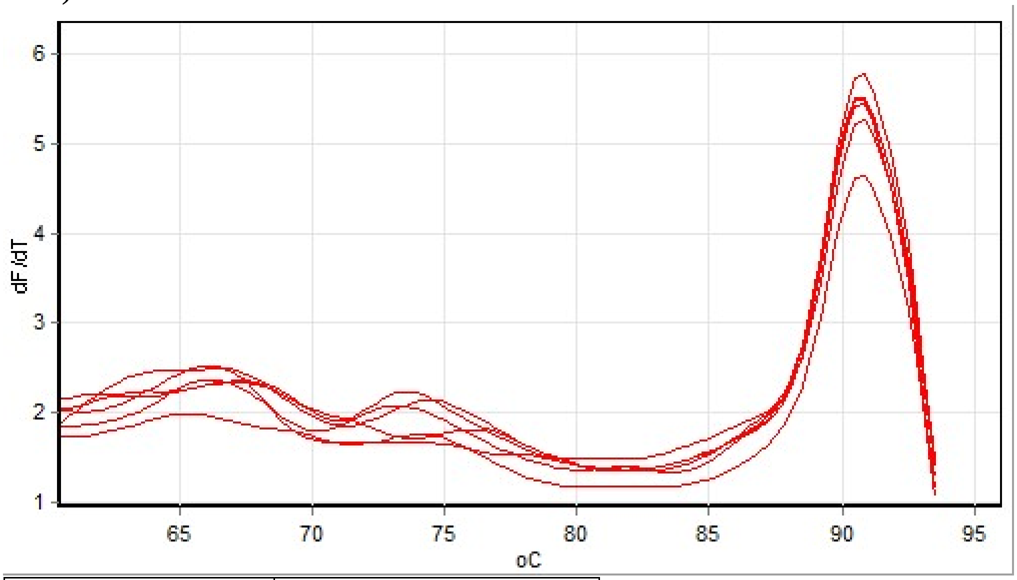

Reaction efficiency (*)	0,91538 (* = 10^(-1/m) - 1)

J) EF2

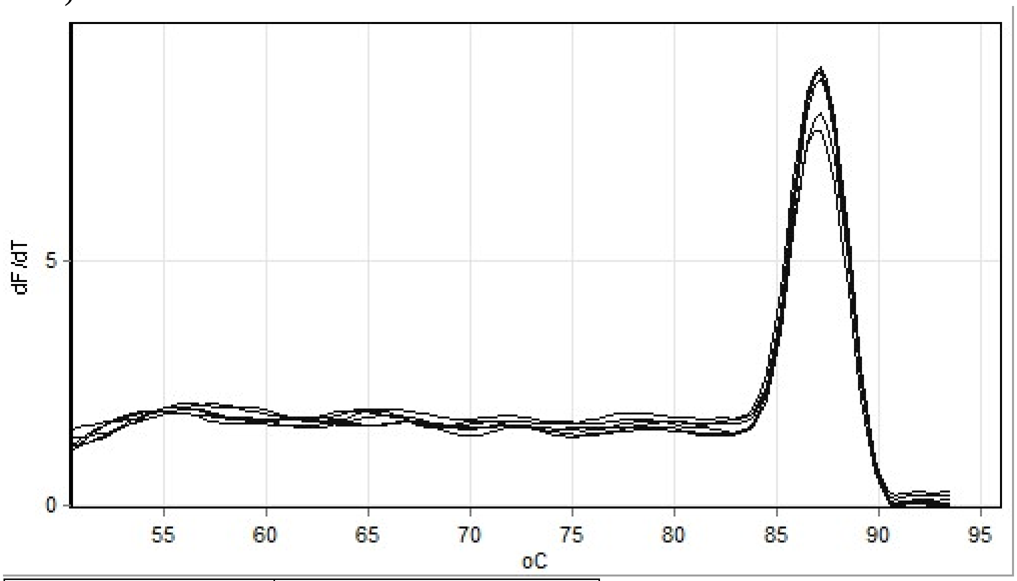

Reaction efficiency (*)	1,01498 (* = 10^(-1/m) - 1)

Fig. S3 Melting curves and reaction efficiencies for primer pairs of *HvIPT* (A-G), *HvCKX1* (H), *HvCKX3* (I) and *EF2* (J) genes. The reaction efficiencies were calculated based on series of standards dilutions of each gene.
